# Capturing dynamic relevance in Boolean networks using graph theoretical measures

**DOI:** 10.1093/bioinformatics/btab277

**Published:** 2021-05-13

**Authors:** Felix M Weidner, Julian D Schwab, Silke D Werle, Nensi Ikonomi, Ludwig Lausser, Hans A Kestler

**Affiliations:** Institute of Medical Systems Biology, Ulm University, Ulm 89069, Germany; International Graduate School of Molecular Medicine, Ulm University, Ulm 89069, Germany; Institute of Medical Systems Biology, Ulm University, Ulm 89069, Germany; Institute of Medical Systems Biology, Ulm University, Ulm 89069, Germany; International Graduate School of Molecular Medicine, Ulm University, Ulm 89069, Germany; Institute of Medical Systems Biology, Ulm University, Ulm 89069, Germany; International Graduate School of Molecular Medicine, Ulm University, Ulm 89069, Germany; Institute of Medical Systems Biology, Ulm University, Ulm 89069, Germany; Institute of Medical Systems Biology, Ulm University, Ulm 89069, Germany

## Abstract

**Motivation:**

Interaction graphs are able to describe regulatory dependencies between compounds without capturing dynamics. In contrast, mathematical models that are based on interaction graphs allow to investigate the dynamics of biological systems. However, since dynamic complexity of these models grows exponentially with their size, exhaustive analyses of the dynamics and consequently screening all possible interventions eventually becomes infeasible. Thus, we designed an approach to identify dynamically relevant compounds based on the static network topology.

**Results:**

Here, we present a method only based on static properties to identify dynamically influencing nodes. Coupling vertex betweenness and determinative power, we could capture relevant nodes for changing dynamics with an accuracy of 75% in a set of 35 published logical models. Further analyses of the selected compounds’ connectivity unravelled a new class of not highly connected nodes with high impact on the networks’ dynamics, which we call gatekeepers. We validated our method’s working concept on logical models, which can be readily scaled up to complex interaction networks, where dynamic analyses are not even feasible.

**Availability and implementation:**

Code is freely available at https://github.com/sysbio-bioinf/BNStatic.

**Supplementary information:**

Supplementary data are available at *Bioinformatics* online.

## 1 Introduction

Given the complexity of biological systems, holistic approaches that tend to describe general dynamic behaviours are required. For this purpose, various modelling approaches ranging from discrete to continuous have been applied. Altogether, these modelling approaches are based on simple interaction graphs depicting interaction partners in complex biological contexts. However, while complex interaction graphs, such as protein–protein interactions networks, are quite large, mathematical models are restricted to a limited number of nodes. This size limitation is based on the information required to build these models. In this context, discrete models as Boolean networks (BNs) (Kauffman, 1969), are less restrictive. Thus they can be constructed and enlarged by both literature and reverse engineering from time series (Hopfensitz *et al.*, 2011; Laubenbacher and Stigler, 2004; Maucher *et al.*, 2011, 2014; Veliz-Cuba, 2012). Nevertheless, also for BNs it holds that dynamic complexity scales exponentially with network size, again limiting the possibility of complete dynamic investigations (Schwab *et al.*, 2020).

**Fig. 1.**
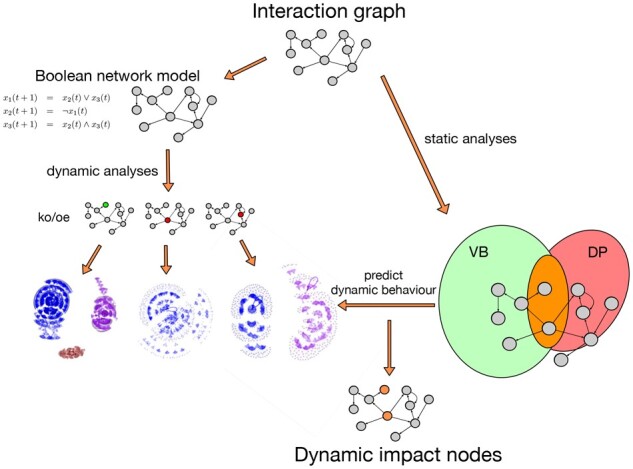
Schematic description of the approach. Using a combination of the two graph-based measures VB and DP on the interaction graph allows to predict dynamic behaviour and nodes most relevant for that (right). Traditional approaches rely on certain dynamic models such as Boolean networks (left). These models are used to simulate the dynamics under various perturbation scenarios such as knock-out or overexpression to determine the most relevant nodes. In contrast, due to the smaller search space, the interaction graph-based approach allows to screen for these nodes more efficiently

Different studies pointed to the possibility that a limited number of nodes are responsible for the dynamic behaviour of a complete network (Jeong *et al.*, 2001). Therefore, we addressed whether it is possible to capture dynamically relevant nodes only by relying on interaction graph properties. In this direction, multiple static measures have been suggested in graph theory (Freeman, 1977; Heckel *et al.*, 2013; Matache and Matache, 2016), still missing analyses on their potential impact in predicting alterations in dynamics. To fill this gap between interaction graph and dynamic analyses, we selected a set of 35 published BNs and used them for identifying dynamic drivers. This decision is based on the fact that BN models have proven to be quite capable of predicting complex biological behaviours (Albert and Othmer, 2003; Dahlhaus *et al.*, 2016; Davidich and Bornholdt, 2008; Herrmann *et al.*, 2012; Ikonomi *et al.*, 2020; Meyer *et al.*, 2017; Siegle *et al.*, 2018; Werle *et al.*, 2021), together with reasonable simulation times for dynamic analyses. Therefore, we first performed feature selection experiments to determine the static measures which are most promising to predict the dynamic behaviour. Then, we evaluated if combinations of well-performing measures were also promising. We obtained a set of nodes only by static investigation to predict dynamic impact (Fig. 1). Finally, we investigated further properties of our selected group of nodes, interestingly individuating a set of nodes which was previously undefined. These nodes that we called ‘gatekeepers’, are not highly connected and show a high impact on the dynamic behaviour.

## 2 Materials and methods

### 2.1 Boolean network models

In BNs, every compound entity *g* is translated to a binary variable $x_{g}\in \{0,1\}$. The corresponding variable describing a compound’s activity is time-dependent and thus $x_{g}=x_{g}(t)$. Furthermore, each compound is assigned a fixed transition function $f_{g}(x_{1},\ldots ,x_{g},\ldots ,x_{n})$ describing its regulations. For a network *N* consisting of *n* nodes in total, activity states change at some point in time and thus are described by a vector of *n* variables $x(t):=(x_{1}(t),\ldots ,x_{n}(t))$. Such a set is referred to as the system’s state. To update the state of a network to the next step in time, the transition functions *f_g_* of all genes have to be evaluated. For this purpose, different updating schemes can be applied (Gershenson, 2004). The most simple one is synchronous updating where all transition functions are evaluated synchronously (Kauffman, 1969), that is for every compound *g*, $x_{g}(t+1)=f_{g}(x_{1}(t),\ldots ,x_{n}(t))$. Due to the finite size of the state space and the deterministic nature of synchronous BNs, each state will lead to a recurring state or set of states. These repeating states are called attractors. In a biological context, attractors can be associated to phenotypes (Kauffman, 1993).

For the analyses, we used a set of published BNs of various biological processes to investigate the ability to predict compounds with high dynamic impact only based on static measures. Extensive literature research was conducted to screen for BN models, as explained in Supplementary Section 1. This has yielded a data set of 35 BNs (Supplementary Table S1) ranging in size from 5 to 51 genes, with an average size of 20 nodes. The median number of nodes in networks is 18, with an interquartile range of 10.5 (from 12.5 to 23). The number of regulations ranged from 12 in a network of 7 nodes to 158 in a network of 32 nodes, with an average of 48 edges for a total number of 684 nodes.

### 2.2 Dynamic measures

We used the R-package BoolNet (Müssel *et al.*, 2010) for simulation of the BNs’ dynamics. Each network *N* was simulated using the synchronous update strategy. The dynamics of a BN can be modified by perturbations. Perturbations such as fixing the state of a node correspond to laboratory overexpression (OE, $x_{g}(t):=1$) or knockout (KO, $x_{g}(t):=0$) experiments. This may lead the system towards a different set of attractors. The dynamic impact of each compound *g* of *N* was determined by the change of the networks’ dynamics after these compounds perturbation (P) (OE or KO, P∈{OE,KO}). The corresponding perturbed network will be denoted by the symbol $N_{g}^{P}$. Measurements of the changes in the dynamics were based on the networks’ set of attractors A(N). An attractor a∈A(N) will be represented as a vector $a=(a_{1},\ldots ,a_{n})$ of trinary variables $a_{g}\in \{0,1,0.5\}$ indicating presence, absence or oscillating behaviour. We additionally use the notation $A_{g}(N)$ to denote attractors without component *g*. For instance, $a\in A_{g}(N)$ correspond to $a=(a_{1},\ldots ,a_{g-1},a_{g+1},\ldots ,a_{n}$).

We applied three different dynamic measures to quantify changes in attractors after perturbing component *g* by P∈{OE,KO}. Using this method allows us to rank the different compounds of each network according to their dynamic impact.

*Gain of attractors* (*G_g_*): The gain of attractors calculates the number of attractors which emerge after perturbation. It returns the number of attractors present in the modified set and not in the original attractors

(1) $$G_{g}=\underset{P}{\max}\left|A_{g}(N_{g}^{P})\setminus A_{g}(N)\right|.$$

Here, the maximal number of newly created attractors is chosen for each gene *g*, whether this be through OE or KO. Over all *n* nodes the gain of attractors is analysed a vector $G={\left(G_{g}\right)}_{g=1}^{n}$.

*Loss of attractors* (*L_g_*): The loss of attractors returns the number of original attractors for which there exists no match in the modified set

(2) $$L_{g}=\underset{P}{\max}\left|A_{g}(N)\setminus \left(A_{g}(N)\setminus A_{g}(N_{g}^{P})\right)\right|.$$

The vector of attractor losses of all *n* nodes is given by $L={\left(L_{g}\right)}_{g=1}^{n}$.

*Minimal Hamming distance* (*D_g_*): The third measure is based on the Hamming distance H(a,a′), which sums up the absolute differences between **a** and a′ for every component (Hamming, 1950). However, the perturbed compound *g* is not incorporated into the distance. Only the effects caused to other compounds by this perturbation are considered $H_{g}(a,a\prime )=\sum_{g\prime \neq g}\left|a_{g\prime }-a\prime_{g\prime }\right|$. *D_g_* quantifies the minimal shift in attractors caused by the perturbation. Here, any given attractor in the perturbed system is compared to the set of all attractors in the original system. *D_g_* measures the Hamming distance to the one which most closely resembles its own pattern of gene expression.

(3) $$D_{g}=\underset{P}{\max}\frac{1}{\left|A(N_{g}^{P})\right|}\sum_{a\prime =A(N_{g}^{P})}\underset{a\in A(N)}{\min}H_{g}(a,a\prime ).$$

The corresponding vector is denoted by $D={\left(D_{g}\right)}_{g=1}^{n}$.

*Dynamic impact* (*I_g_*): The three measures given above can be aggregated to one single measure for the dynamic impact of the individual node $I=\frac{1}{3}\left(\text{rk}\left(G\right)+\text{rk}\left(L\right)+\text{rk}\left(D\right)\right),$ where rk(.) denotes the ranking function. Here compounds *g* in a network *N* can be ranked according to the relevance of their perturbation on the dynamics. The dynamic impact *I_g_* of an individual compound *g* can now be extracted from $I={\left(I_{g}\right)}_{g=1}^{n}$.

### 2.3 Static measures

We investigated a range of graph-theoretical measures that assign individual values to each node according to the specific properties of networks’ topology. In contrast, dynamic properties are derived from the state graph which grows exponentially with the number of compounds in the system. Due to the smaller search space, measures of static properties can be calculated on a faster time scale than their dynamic counterparts. We performed feature selection experiments to determine the static measures, which are most promising to predict the dynamic behaviour. The classification was done using all possible combinations of the static interaction graph-based features over all nodes of all networks *N*. Each node *g* was labelled with its dynamic impact measured by *I_g_*. For classification, we used k-Nearest-Neighbour, random forest, and support vector machine algorithms with linear and radial basis function kernels. Classification performance was measured using 10x10 and leave-one-subset-out (leaving out one network) cross-validation (CV). For the 10 × 10-CV, results show a CV accuracy ranging between minima of 0.461 and 0.545 across algorithms and maxima between 0.691 and 0.749 over all possible feature combinations. Analogously, the reclassification accuracy ranges between minima of 0.469 and 0.569 to maxima between 0.705 and 1.0. The two static measures vertex betweenness and determinative power are overrepresented over the best performing feature combinations. Thus, we considered them as the most promising features for further evaluation (see detailed descriptions of methods and results in Supplementary Section 2).

*Interaction graphs* (I): The interaction graph I=G(V,E) contains a set V of *n* nodes representing genes as well as other components or processes of the system. These are connected via directed edges E which represent regulatory influences. That is, an edge $(g_{i},g_{j})\in E$ pointing from gene *g_i_* to gene *g_j_* indicates the presence of *g_i_* in the Boolean transition function of *g_j_*. The interaction graph therefore captures only (static) topological properties of the system.

*Vertex betweenness* (*VB_g_*): The vertex betweenness, or shortest-path betweenness (Freeman, 1977), is derived from the interaction graph I=G(V,E) and can be calculated for every vertex g∈V (i.e. node), each corresponding to a given gene. It analyses to the distribution of shortest paths between nodes. The definition of vertex betweenness *VB_g_* is based on the set of shortest paths *s_ij_* between nodes *g_i_* and *g_j_*, $g_{i}\neq g_{j}$, and its subset $s_{ij}(g)$ of paths which pass node $g\;VB_{g}=\sum_{g_{i},g_{j}\in V\setminus \{g\}}\frac{\left|s_{ij}(g)\right|}{\left|s_{ij}\right|}.$ The more often *g* is passed by the shorted paths the higher is its final score. Similar to the dynamic measures the vector of all *n* vertex betweenness will be denoted as $VB={\left(VB_{g}\right)}_{g=1}^{n}$.

*Determinative power* (*DP_g_*): Determinative power relates to network entropy and assigns high scores to a node *g* if knowledge about the state of this node yields a high ’gain of information’ about the state of its output nodes, as defined in terms of mutual information (MI) (Heckel et al., 2013; Matache and Matache, 2016). The determinative power *DP_g_* of a node *g* is defined by utilizing a measure of the binary Shannon entropy $h(p_{g})=-p_{g}{\;\text{log}\;}_{2}(p_{g})-(1-p_{g}){\;\text{log}\;}_{2}(1-p_{g})$, where $p_{g}=P(X_{g}=1)$ describes the probability of a random, binary variable *X_g_* taking the value *x_g_* = 1 (Matache and Matache, 2016).

Furthermore, the support of a given Boolean regulation function *f_g_* is defined as the set of states which are mapped to an output of 1 by the function, formally described as $S(f_{g})=\{x:f_{g}(x)=1\}$.

The reduction of uncertainty about the values of genes in the support of *f_g_* (Climent *et al.*, 2010) achieved by knowledge of the state of node g′ is described using MI.

(4) $$\begin{array}{c} MI(X_{g};f_{g\prime }(X))=h\left(\sum_{x\in S(f_{g\prime })}p_{x}\right)-\\ \sum_{b\in \{0,1\}}P(X_{g}=b)h\left(\sum_{x\in S(f_{g\prime })}P(X=x|X_{g}=b)\right). \end{array}$$

Here $p_{x}$ denote the probability of state **x** and $X={\left(X_{g}\right)}_{g=1}^{n}$.

Finally, this yields the determinative power of node *g* as *DP_g_*, summing over all outputs g′ of node *g* $DP_{g}=\sum_{g\prime =1}^{n}MI(X_{g};f_{g\prime }(X))$. As the value of $MI(X_{g},f_{g\prime }(X))$ is maximally one, the values of the determinative power of a node ranges between zero and the number of outputs of the node (Pentzien *et al.*, 2018). The corresponding vector of determinative power will be denoted as $DP={\left(DP_{g}\right)}_{g=1}^{n}$.

### 2.4 Gene impact ranking

In the following, different selection sets are obtained from various combinations $VB_{T}$ and $DP_{T}$, the top scoring genes of the two static measures. Here T∈[1%;100%] denotes the percentage of all *n* genes. More precisely, $VB_{T}$ denotes the top scoring gene set

(5) $$VB_{T}=\left\{g:r_{g}\leq \left\lceil Tn\right\rceil ,r=rk(VB)\right\},$$

where $r={\left(r_{g}\right)}_{g=1}^{n}$. $DP_{T}$ is defined analogously. We analyse $VB_{T},\;DP_{T}$, the union $VB_{T}\cup DP_{T}$, and the intersection $VB_{T}\cap DP_{T}$ for their intersect with the top dynamic impact genes $I_{T}$. To investigate whether a stricter or broader criterion should be chosen for labelling a gene as having high impact the comparison between statics and dynamics is conducted for all possible sizes T∈[1%;100%] of the selected set. Based on this threshold, genes are classified as either high or low impact according to their scores on the presented static and dynamic measures individually.

### 2.5 Connectivity of nodes

Connectivity of compounds in the interaction graph is considered to be relevant to determine high-impact nodes (Guimera and Amaral, 2005). Connectivity is quantified using z-scores *C_g_* for a given gene *g*, where *δ_g_* is the total degree of node *g*, while $\bar{\delta }$ is the average total degree of all nodes in the network. *σ* is the corresponding standard deviation of these total degrees in the same network $C_{g}=\frac{\delta_{g}-\bar{\delta }}{\sigma }$. The corresponding vector is denoted as $C={\left(C_{g}\right)}_{g=1}^{n}$.

A hub is then defined as a node having a z-score in connectivity of $C_{g}\geq 2.5$, as given by (Guimera and Amaral, 2005). Hubs are considered to be master regulators of biological processes (Borneman *et al.*, 2006; He and Zhang, 2006).

## 3 Results

Formal descriptions of regulatory interactions of a system can be represented by different types of mathematical models, and can allow for the investigation of dynamics. However, since dynamic complexity grows exponentially with size, dynamic investigation is limited. In contrast, the size of static interaction graphs only grows linearly when adding components. Therefore, it is of some interest to find methods based on interaction graph properties to capture dynamic drivers. Such a method would finally reduce complex dynamic analyses, as a screening with static-based methods can drastically reduce the search space for dynamic analysis. This procedure allows finding dynamic influencing compounds and, thus, potential target candidates on large models. On these grounds, we considered BNs, as best candidates to evaluate our approach.

### 3.1 Combination of static measures captures dynamic influencing compounds

We considered a range of eight commonly described static measures, namely VB (Freeman, 1977), DP (Heckel et al., 2013; Matache and Matache, 2016), connectivity (Guimera and Amaral, 2005), resistance distance (Klein and Randić, 1993), coreness (Giatsidis *et al.*, 2013), eigenvector centrality (Newman, 2008), eccentricity (Hage and Harary, 1995) and shimbel index (Rodrigue, 2016; Shimbel, 1951, 1953). By applying feature selection, we compared their performance in predicting the dynamic characteristics defined in our method sections (Supplementary Section 2). By training on 35 published BN models, VB and DP performed best in predicting dynamic relevant compounds (Supplementary Figs S1 and S2).

**Fig. 2.**
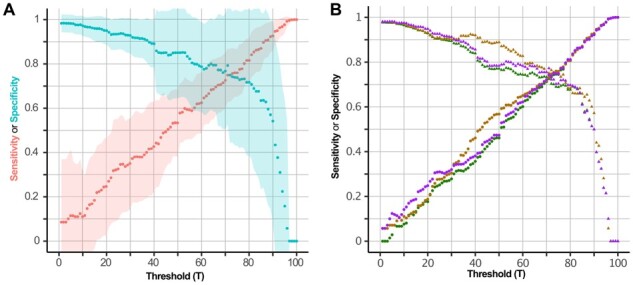
Static measures to determine important dynamic nodes. (**A**) The figure shows the sensitivity (red) and specificity (blue) yield by the comparison of static measures (vertex betweenness and determinative power) and the average of the three dynamic parameters at each threshold *T* = {1,…,100} over all networks. Dots represent the average sensitivity and specificity and coloured regions display the standard deviations. Here, the point of intersection between sensitivity and specificity is at 0.756 corresponding to a threshold *T* = 73%. (**B**) The performance of the intersection against single dynamic measures is depicted at each threshold *T* = {1,…,100}. The figure shows that considering single dynamic measures (Hamming distance in purple, attractor loss in brown, attractor gain in green) is comparable to using their average value

Next, we considered the possibility that combinations of these two measures might improve the prediction power of dynamic features. Hence, selected nodes by VB, DP, their intersection, and their union were considered, and their specificity and sensitivity was evaluated at different thresholds *T*. To do so, each node was perturbed and three dynamic measures have been computed and averaged for each given threshold *T*. Among the static measures, $VB_{T}\cap DP_{T}$ yielded the best results considering both single dynamic measures and their average (Fig. 2A and B, Supplementary Figs S3 and S4). Here, we obtained a sensitivity of 0.756 at a threshold of 73% with an accuracy of 0.756 ± 0.136 for all 35 networks. Furthermore, the selection threshold stability has been successfully addressed by bootstrap analysis (Supplementary Fig. S5), as well as the independence of the method towards network size (Supplementary Fig. S6). Finally, these results confirm that there actually exists a combination of static measures able to faithfully capture changes in dynamic behaviours and consequently their drivers. Thus, these measures are of high potential to reduce the simulation complexity and consequently allow for faster predictions and more complex models. This method provides a fast and easy approach to select relevant dynamic drivers in any type of interaction graph-based method.

### 3.2 Characterization of the selected compounds reveals a new class of dynamic drivers

To further characterize the selected set of compounds, we started from the shared concept that highly connected compounds strongly influence dynamic behaviour (Jeong et al., 2001). Hence, we considered to study the relationship between connectivity and dynamic influence. First, we observed that highly connected nodes (hubs) were all selected by our method, further corroborating its correctness. However, according to the definition of hub nodes (Guimera and Amaral, 2005) only 21 compounds (3.1% of the selection) were classified as highly connected. Therefore, we considered that among our selection, other statically definable classes of dynamic influencing nodes might exist. To address this possibility, we deepened our analyses measuring mismatches between the impacts regarding connectivity and our approach. The overall results of our approach, the selection given by the $VB_{T}\cap DP_{T}$ at T=73%, selected 424 nodes out of 684 in total. Nodes excluded from this first selection are 260 (38.0%). This group we named non-selected (NS). To further investigate the set of selected nodes, we ranked them by (i) their static impact according to VB and DP as an average, and (ii) their connectivity. For all nodes in $VB_{T}\cap DP_{T}$ at T=73%, we considered mismatches between these two rankings. Therefore, we divided our nodes as follows: Positive mismatches are nodes that have a higher average ranking in DP and VB than in connectivity. Negative mismatches instead are nodes with lower or equal average ranking of VB and DP compared to connectivity. As a result, the aim of our analysis is to investigate whether it is possible to find nodes with high static and dynamic impact, which are also not highly connected.

**Fig. 3.**
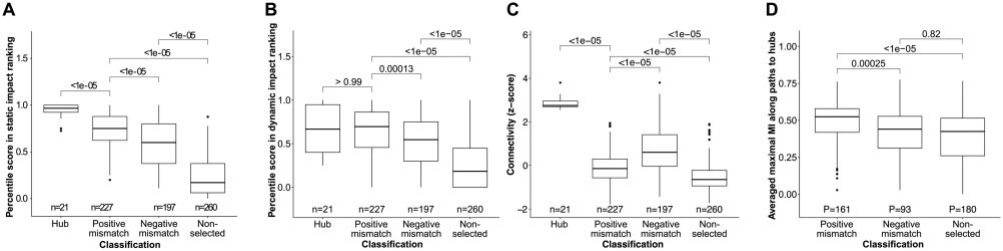
Characterization of selected nodes. (**A**) Percentile scores of static impacts. Selected nodes from the intersection of the two static measures vertex betweenness and determinative power are further divided in hubs, positive mismatches and negative mismatch nodes (n). Hub nodes are shown to have a higher percentile score in static ranking. (**B**) Impact on dynamic ranking. The percentile score in dynamic impact for each of the selected and non-selected subgroups is depicted. Hubs and positive mismatch nodes have comparable dynamic impact whereas negative mismatches and none selected have significantly reduced impact. (**C**) Connectivity defined by the z-score is depicted for the selected and none selected subgroups. Positive mismatches show significantly lower connectivity compared to hubs and negative mismatches. (**D**) Average maximal mutual information (MI) in paths (p) to hubs. Positive mismatch nodes show a significantly higher MI then negative mismatches and not selected nodes. Statistical tests were performed using a Bonferroni corrected Wilcoxon rank sum test. Significant values are considered for *p *<* *0.05

Out of all 684 nodes across all networks, 227 (33.2%) are classified as positive mismatch. The remaining 197 (28.8%) selected nodes are classified as negative mismatches (Fig. 3). Positive mismatch nodes have a lower score in static impact ($p<1\cdot 10^{-5}$), if compared to hub nodes. However, this group of nodes has a dynamic impact comparable to the one of hubs (*p *>* *0.99) and significantly higher compared to other groups (*p *<* *0.0001) (Fig. 3A and B). Accordingly, for hubs, the median for the dynamic impact is at 0.667, whereas for positive mismatch nodes the median is 0.696. Negative mismatch nodes have a median of 0.546. Moreover, positive mismatch nodes show significantly lower connectivity if compared to hubs and negative mismatching nodes ($p<1\cdot 10^{-5}$, Fig. 3C). Our results unravelled the existence of a group of nodes characterized by low connectivity and high dynamic impact. Interestingly, this group can be identified only based on topological measures.

Next, we compared if this set of selected nodes and their connectivity-based subgroups can be identified also by other published interaction graph-based methods. To do so, we considered three static features that have been linked to control of dynamic behaviours: Canalysing variables (Murrugarra and Dimitrova, 2015), feedback vertex set (FVS) (Zañudo *et al.*, 2017), and network motifs (Milo *et al.*, 2002). Thus, we investigated nodes which act as canalysing variables (Murrugarra and Dimitrova, 2015), nodes belonging to the FVS of a network (Zañudo et al., 2017), as well as nodes which participate in network motifs (Albergante *et al.*, 2014; Milo et al., 2002). An important feature of biologically motivated networks is the presence of canalysing functions. Multiple levels of canalysation can be described, depending on the number of functions which a node is canalysing. In total, we could identify 543 (79.4%) of all nodes present in the set of analysed BNs acting as canalysers (see Supplementary Table S3). Out of these, 73.9% belong to the set $VB_{T}\cap DP_{T}$. Furthermore, there is any particular enrichment of canalysing nodes in neither the positive or the negative mismatch subgroups. Moreover, by increasing the level of canalysation the distribution into the subgroups of the canalysing nodes shifts towards the negative mismatch class (Supplementary Table S3). This results from the fact that highly canalysing nodes are also more highly connected. Similarily to canalysing nodes, biological networks show occurrence of certain network motifs (Milo et al., 2002). Albergante et al. (2014), could show that short feed forward loops are particularly affecting the stability of protein–protein interaction networks. Therefore, we investigated the frequency of participation of nodes in coherent and incoherent feedforward loops (C1FFL and I1FFL), as well as the bifan motif. Among our investigated BNs we found a total of 1086 C1FFLs, 112 I1FFLs and 1197 bifans. Around 80% of nodes that participate in each of these motifs fall also in the selected $VB_{T}\cap DP_{T}$ (Supplementary Fig. S7). However, again, we could not observe any particular enrichment in one of our identified subgroups of positive and negative mismatches. Finally, the FVS is described as group of nodes that can be used to control BNs dynamics (Zañudo et al., 2017). In total, by a network-based analysis, we could detect 138 (20.2%) nodes identified as FVS. Again, taking all nodes into account, around 83% of the FVS is also selected by $VB_{T}\cap DP_{T}$ (Supplementary Fig. S8). These results lead us to the conclusion that our method selects a unique set of nodes defined by $VB_{T}\cap DP_{T}$, that is not a subset of any known previously described static features. Even more interestingly, the class of positive mismatches was confirmed not to be univocally detectable by any of these methods. Thus, we conclude that we actually identified a completely new class of nodes.

### 3.3 Higher mutual information characterizes positive mismatches nodes as gatekeepers of hubs

In the previous sections, we showed that $VB_{T}\cap DP_{T}$ yields a set of nodes that can capture dynamic relevant changes in a selection of 35 BNs. Further, we showed that this selected set can be further be sub-grouped as positive and negative mismatches. We could show that these groups are uniquely identified by our method and not by others. Since positive mismatches show the unique feature of being not highly connected and still highly impacting dynamic behaviour, we further characterized this newly identified class of compounds. Combining the idea of hubs as master regulators of biological processes, together with the possibility of new groups of dynamic drivers arising, we hypotheses that the positive mismatch class is somehow affecting dynamics by acting through hubs. To evaluate this concept, we considered the reliability with which signals are passed among these nodes. All simple paths (i.e. paths in which no vertex is visited twice) across networks starting at a non-hub and ending at a hub node are calculated and compared depending on the classification of the start node. The MI as given by equation (4) was calculated for every edge along these paths and normalized by the number of edges. Every path *P* is thus assigned a value $MI_{p}\in [0,1]$. Across all pairs of non-hub starting nodes and hub end nodes, the maximal MI values among their connecting paths are compared. The distribution of these values by group across networks is shown in Figure 3D. Here, we could show that there exist channels of information flow beginning at positive-mismatch nodes and more reliably affecting hubs. This might indicate a special gatekeeper role of these positive-mismatch nodes. Hence, we will refer to this new group of nodes as gatekeepers. Given that BN models depict parts of complex biological regulations, input nodes are often used to trigger activation or inhibition of cascades. However, these nodes are considered as external inputs and not regulated within the network. Hence, we would not expect input nodes to have a major impact in our analysis. In fact, the majority of input nodes across networks fall into the low-impact group of NS genes. For a total of 53 input nodes, 44 (83.0%) are classified as NS, while 7 (13.2%) are assigned to the positive-mismatch group and 2 (3.8%) to the negative mismatch group.

Having shown that there actually is an information flow between hubs and gatekeepers, we investigated the potential nature of this relationship. One hypothesis in this sense, could be that these two classes of nodes are co-expressed. To address this idea, we performed the co-expression analysis available in STRING (Szklarczyk *et al.*, 2016). Our results show that gatekeepers and hubs seem not to be significantly co-expressed (Supplementary Table S4). Another important relationship between compounds is mutual exclusivity, which is relevant especially in disease development and treatment (Völkel *et al.*, 2020). Therefore, we investigated mutually exclusive pairs of hubs and gatekeepers for human networks using cBioPortal (Gao *et al.*, 2013) (Supplementary Table S5). Again, we did not find enrichments of significantly mutually exclusive couples of hubs and gatekeepers. Altogether, even if we could show a significant exchange of MI between hubs and gatekeepers, still further investigations will be required to elucidate how this information is exactly transferred.

### 3.4 Perturbation of gatekeepers impacts biological phenotypes

In the previous sections, gatekeeper nodes were identified and described. Next, it is evaluated whether perturbations of gatekeeper nodes can biologically impact phenotypes of investigated networks. For this purpose, interventions on gatekeepers were compared with experimental results. The BN by Cohen *et al.* (2015) depicts pathways involved in tumour development by leading to invasion and metastases. In the network one gatekeeper node, twist-related protein 1 (TWIST1), is of particular interest. *In silico* KO of TWIST1 impairs tumoural associated behaviour. In accordance, TWIST1 KO in breast cancer cells inhibits the expression of epithelial to mesenchymal transition (EMT) markers, preventing metastases formation in immune-deficient mice (Li *et al.*, 2014; Xu *et al.*, 2017). Furthermore, a similar effect of TWIST1 on invasion potential has been observed also in other types of tumours, such as prostate cancer, melanoma, and glioblastoma (Cho *et al.*, 2013; Mikheeva *et al.*, 2010; Weiss *et al.*, 2012). Another example of the biological impact of not highly connected gatekeepers is the network of Méndez-López *et al.*, (2017). Also here, the network describes the EMT process. Again, individuated gatekeepers [E74 Like ETS Transcription Factor 5 (ESE2) and cyclin-dependant kinase inhibitor 2A (p16)] are not hub nodes. The unperturbed network simulation leads to three single state attractors describing epithelial, senescent and mesenchymal characteristics (Méndez-López et al., 2017). The highest on attractor changes after perturbation can be observed in the context of ESE2 loss of function, leading to an attractor with only mesenchymal characteristics. In accordance, experimental results in ESE2 conditional KO mice show induction of EMT by upregulation of Snail Family Transcriptional Repressor 2 (Snail2) (Chakrabarti *et al.*, 2012). Similar results have been shown for loss of ESE2 in both breast and prostate cancer, identifying also a prognostic value linked to its expression in cancer tissues (Feldman *et al.*, 2003; Li *et al.*, 2017; Watson *et al.*, 2010; Yao *et al.*, 2015). Besides tumour associated phenotypes, we also investigated the effect of gatekeepers in homeostatic systems. In this context, the network of Krumsiek *et al.* (2011) describes differentiation decision-makings in the hematopoietic system. Here, the authors describe loss of hematopoietic phenotypes concomitant with KO of identified gatekeepers such as GATA binding protein 2 (GATA2), friend leukaemia integration 1 (FLI1), CCAAT enhancer binding protein alpha (CEBPA), Spi-1 proto-oncogene (PU1) and growth factor independence 1 (GFI1). Altogether, we could show that alterations of gatekeeper nodes majorly affect biological phenotypes connected to both disease and physiological conditions.

## 4 Discussion

There is a variety of proven approaches to identify nodes of high impact on the dynamics of regulatory networks (Gonzalez *et al.*, 2006; Klamt *et al.*, 2007, 2006; Paulevé, 2017; Schwab *et al.*, 2016; Zheng *et al.*, 2010). Each of the mentioned approaches relies on the determination of intervention targets by calculation of the networks’ dynamics. In contrast, our method is based only on static properties of nodes in interactions graphs. Thus, it can be applied to a large variety of mathematical models, or even simpler, to interaction graphs. To validate our approach, BNs were used as reference models. This choice is driven by the biological relevance of these models, together with their ability to cover a wide range of network sizes and the availability of these models.

Here, we set up a selection method based on interaction graph properties by studying the dynamic and static features of 35 published BNs. We could show that the intersection of two static measures, VB and DP, can faithfully capture dynamic influencing nodes. On the one hand, by comparing the selected set of nodes from our approach to the ones of other graph-based measures (Milo et al., 2002; Murrugarra and Dimitrova, 2015; Zañudo et al., 2017), we could find a wide overlap of nodes compared to each of the other approaches. This indicates that the intersection of VB and DP is actually selecting a relevant subset of nodes. On the other hand, results show the method uncovers additional relevant nodes. Additionally, applying the method provides a series of advantages. First, the static selection according to VB and DP can be applied very broadly. Methods based on detecting canalysing nodes, instead, require canalysing functions (Murrugarra and Dimitrova, 2015). Methods like FVS provide sets of nodes for control of networks. However, the whole set needs to be controlled in order to shift the dynamic behaviour. This is not the case here: each of the nodes selected by the static intersection can yield a dynamic shift when perturbed. Due to this fact, the method can also be applied for e.g. drug targeting purposes, or to detect dynamic drivers. In this context the method can have multiple benefits. On the one side, a limited amount of druggable targets is desirable in clinical settings (Palmer and Sorger, 2017). On the other, it has been shown that disease drivers are single or combined alterations that then cause disrupted cellular signalling (Völkel et al., 2020). In addition, our selection can be further subdivided into two subgroups: positive and negative mismatches. In particular, the positive-mismatch subgroup of the selection provides a new set of compounds with high dynamic impact while not being highly connected. For measuring connectivity, we applied the commonly used z-score of in- and output degree normalized by mean and standard deviation across the whole network (Chee and Byron, 2021; Fiscon *et al.*, 2018; Li Mow Chee and Byron, 2021). Another, more robust definition of the z-score is based on the median and median absolute deviation (Rousseeuw and Hubert, 2011). In addition to the standard z-score, we also based our classification into PM, NM, and NS on this robust z-score. Our results show that the classification of nodes remains stable using this robust z-score (see Supplementary Table S2). This subset of nodes cannot be detected univocally by any previously published method (Supplementary Fig. S9). Instead, these other methods (Milo et al., 2002; Murrugarra and Dimitrova, 2015; Zañudo et al., 2017) tend to select the class of negative mismatch compounds. This means, these methods tend to select highly connected nodes with high dynamic impact. Hence, the selection captures two types of compounds based on static properties. First, the well-known and detectable highly connected dynamic influencing drivers. Second, a new set of dynamic drivers, which we called gatekeepers—nodes with high dynamic relevance but no high connectivity. Notably, the majority of input nodes (86.8%) do not fall into this new class. Moreover, none of the hub nodes was present in the class of gatekeepers. Based on our results, we hypothesize that the high dynamic impact of a new class of gatekeeper nodes may arise due to perturbations of these nodes being more reliably passed on to downstream hubs. This hypothesis would be corroborated by the implications of nodes having a disproportionately high DP or VB. A node ranking high in DP indicates a higher likelihood that the effect of modifications will influence a nodes’ direct outputs and will not be outweighed by the influence of other inputs of these target genes. The existence of paths from gatekeeper nodes to hubs having a higher maximal MI than those of other classes further demonstrates that this principle extends to longer paths, that is there exist channels of information flow which are more stable carriers of signals. High values of VB further suggest that a node represents a bottleneck in the communication between various modular subnetworks (Yu *et al.*, 2007). A perturbation of a high-VB node should, therefore, have a disproportionate impact on the dynamic behaviour of genes in a given subnetwork. Ravasz *et al.* (2002) put forth the idea of hierarchical organization in biological networks. This hierarchical organization also indicates that, next to hubs, there are additional nodes on other levels of connectivity which still impact the system’s phenotypes.

Besides theoretical hypotheses, we also investigated the biological co-expression, co-occurrence and mutual exclusivity between gatekeepers and hubs (see Supplementary Tables S4 and S5). Here, we could not find enriched pairs in any scenario. However, this finding may be correlated with time and localization effects of hub interactions. Relating to this, Han *et al.* (2004) subdivided the class of hubs into ’party’ hubs which interact with most of their partners simultaneously and ’date’ hubs, which bind their different partners at different times or localizations. This would indicate that also interactions are process and context-dependent, and may not be detected by analyses based on overall expression data. To further evaluate the importance of gatekeeper nodes, we investigated their biological relevance in three case studies. Results underline the impact of gatekeeper nodes. The low connectivity of these gatekeepers relative to other nodes in the system was confirmed by comparing their connectivity in BNs with the ones from BioGrid (Stark *et al.*, 2006). This characteristic is of fundamental interest in the context of selecting potential intervention targets. Removal of hub nodes correlates with lethal phenotypes (Borneman et al., 2006; He and Zhang, 2006; Jeong et al., 2001), and hubs are difficult to target (Dufour *et al.*, 2013; Lu *et al.*, 2007; Song *et al.*, 2017).

## 5 Conclusion

Here, we presented a new method to identify dynamic affecting compounds based only on static interaction graph properties. The approach aims to rapidly screen interaction networks for their dynamic drivers and, consequently, potential interventions candidates. Furthermore, besides observing considerable overlaps to other methods, our selection strategy could identify a new class of compounds previously unreported. The newly identified gatekeepers provide promising targets for drug selection and disease drivers. Finally, our approach is easily scalable to large directed-graph investigations.

## Funding

H.A.K. acknowledges funding from the German Federal Ministry of Education and Research (BMBF) e: MED confirm (id 01ZX1708C) and TRANSCAN VI—PMTR-pNET (id 01KT1901B). Furthermore, HAK acknowledges funding from the German Science Foundation (DFG, 217328187 (SFB 1074) and 288342734 (GRK HEIST)).

*Conflict of Interest*: The authors declare that there is no conflict of interest.

## Supplementary Material

btab277_Supplementary_Data
