# Supplementary material for: Capturing dynamic relevance in Boolean networks using graph theoretical measures
Source: Bioinformatics. 2021 May 13;37(20):3530–7. doi: 10.1093/bioinformatics/btab277 (PMC8545349; doi:10.1093/bioinformatics/btab277)
Supplement: btab277_Supplementary_Data [file btab277_supplementary_data.pdf]

Supplementary information to:

Capturing dynamic relevance in Boolean networks using graph theoretical measures

1 Network selection

The following table provides references for all 35 networks used in our analyses, as well as their size in number of nodes and edges.
These networks were extracted from the cell collective website (*https://cellcollective.org/*, (Helikar *et al.*, 2012)) as well as from pubMed by using the search term Boolean network model". Thereby, we received a list of 457 items (status 24.05.2017). Networks were discarded if they matched one of the following criteria:
First, networks were checked to have a scale-free degree distribution by using the PoweRlaw R-package (Gillespie, 2015). This package measures the goodness of fit to a power-law distribution. Networks are considered to be scale-free if this p-value is larger than 0.1 (Clauset *et al.*, 2009), otherwise they are discarded. For the calculation of degree distributions, self-loops were counted as single interactions. Furthermore, networks were excluded if it was not feasible to simulate the dynamics in reasonable time or if the network dynamics depend only on its input nodes. Time delays in networks were replaced by dummy nodes.

Authors and Year	Number of nodes	Number of edges
(Azpeitia *et al.*, 2013)	14	24
(Brandon *et al.*, 2015)	22	40
(Calzone *et al.*, 2010)	28	47
(Cohen *et al.*, 2015)	32	158
(Dahlhaus *et al.*, 2016)	23	47
(Davila-Velderrain *et al.*, 2015)	13	42
(Enciso *et al.*, 2016)	26	81
(Fauré *et al.*, 2006)	10	35
(García-Gómez *et al.*, 2017)	16	39
(Giacomantonio and Goodhill, 2010)	5	14
(Gupta *et al.*, 2007)	16	22
(Herrmann *et al.*, 2012)	15	38
(Irons, 2009)	18	59
(Klamt *et al.*, 2006)	40	58
(Krumsiek *et al.*, 2011)	11	30
(MacLean and Studholme, 2010)	7	12
(Mai and Liu, 2009)	41	76
(Marques-Pita and Rocha, 2013)	17	32
(Martinez-Sanchez *et al.*, 2015)	12	72
(Méndez and Mendoza, 2016)	22	44
(Méndez-López *et al.*, 2017)	9	34
(Mendoza and Xenarios, 2006)	23	38
(Meyer *et al.*, 2017)	51	98
(Orlando *et al.*, 2008)	9	19
(Ortiz-Gutiérrez *et al.*, 2015)	14	66
(Ríos *et al.*, 2015)	19	79
(Saadatpour *et al.*, 2011)	18	43
(Sahin *et al.*, 2009)	20	52
(Sankar *et al.*, 2011)	20	30
(Siegle *et al.*, 2018)	23	55
(Sridharan *et al.*, 2012)	19	31
(Sun *et al.*, 2014)	8	13
(Thakar *et al.*, 2012)	33	79
(Todd and Helikar, 2012)	20	46
(Yousefi and Dougherty, 2013)	10	24

Table S1: Authors of the 35 selected networks, including number of nodes and edges in the model.

2 Preliminary analysis of static measures and model selection

In a preliminary analysis, 8 different static measures have been investigated for their ability to predict nodes with high dynamic impact.
In addition to vertex betweenness (VB, (Freeman, 1977)) and determinative power (DP, (Heckel *et al.*, 2013; Matache and Matache, 2016)) (both descirbed in the main manuscript), we evaluated total node degree (Connectivity, (Guimera and Amaral, 2005)), resistance distance (RD, assigning a node the average of resistances to all other nodes (Klein and Randić, 1993)), coreness (kC, i.e. k-core, (Giatsidis *et al.*, 2013)), eigenvector centrality (evc, (Newman, 2008)), eccentricity (ecc, (Hage and Harary, 1995)) and the Shimbel index (si, (Shimbel, 1951, 1953; Rodrigue, 2016)).
These measures as calculated as follows for a network of n nodes:
A is the adjacency matrix of the interaction graph, which has an entry of 1 at index (i, j) if node j is regulated by node i and 0 otherwise.

Connectivity:
The connectivity (see e.g. (Guimera and Amaral, 2005)) sums up the output and input degree of a node. Self-loops are only counted once.

$$\text{Connectivity}(v) = \sum_i A_{i,v} + \sum_i A_{v,i} - A_{i,i} \tag{1}$$

Resistance distance:
The resistance distance of nodes in a network can be obtained from the Laplacian matrix L and the adjacency matrix A (Klein and Randić, 1993). L is obtained as the difference of the degree matrix D and A. The degree matrix contains the total degree of node i at index (i, i) and 0 otherwise.
This matrix can be inverted to obtain the symmetric resistance distance matrix R, which describes how much resistance there is to the flow of information between nodes v and v'. These values are averaged for every node.

$$\begin{aligned} L &= D - A \\ R_{v,v'} &= L^{-1}_{v,v} + L^{-1}_{v',v'} - L^{-1}_{v,v'} - L^{-1}_{v',v} \\ \text{RD}(v) &= \frac{1}{n} \sum_{v'} R_{v,v'} \end{aligned}$$

k-cores:
A node is in the k-core of a graph if it has at least k inputs and k outputs. The highest k for which this is fulfilled is chosen (see (Giatsidis *et al.*, 2013) for further work expanding on this measure).

$$\text{kC}(v) = \min\left(\sum_i A_{i,v}, \sum_i A_{v,i}\right) \tag{2}$$

Eigenvector centrality:
The values of this measure are obtained by solving for the eigenvectors x belonging to the largest eigenvalue λ, using the adjacency matrix for the corresponding undirected graph of the network (Newman, 2008).

$$\begin{aligned} \text{evc}(v) &= \frac{1}{\lambda} \sum_{v'} A_{v,v'} \text{evc}(v') \\ &\Rightarrow Ax = \lambda x \end{aligned}$$

Eccentricity:
The eccentricity of a node is its shortest path distance minlength from the farthest other node in the graph (Hage and Harary, 1995).

$$\mathrm{ecc}(v) = \max_{v' \in \{1,\ldots,N\}} (\mathrm{minlength}_{v \to v'}) \tag{3}$$

Shimbel Index:
The Shimbel Index of a node is given by the sum of lengths of shortest paths minlength from node v to all reachable nodes, given by the number of nodes in these paths, as used by e.g. (Shimbel, 1951, 1953; Rodrigue, 2016).

$$\mathrm{si}(v) = \sum_{v'} \mathrm{minlength}_{v \to v'} \tag{4}$$

Like the 3 presented dynamic measures and their average, all of these static measures assign values to every node in a network.
The dynamic measure of Hamming distance is normalised by the number of nodes in the network while the measure of attractor gain is normalised by the number of attractors in the unperturbed network. For the measure of attractor loss, the fraction of retained attractors is used which is thus in the range [0,1].
For static measures, the values for connectivity, eccentricity as well as the Shimbel Index have been normalised by the number of nodes in their respective network in order to make these measures more comparable across networks of different sizes.
In total, there are 684 nodes across 35 networks. For every fold in every run of e.g. a 10x10 cross-validation, a test set is determined which is then separated from the remaining training set. All static and dynamic measures across all nodes are then transformed into z-scores, using the mean and standard deviation across nodes in the training set for normalisation. The z-scores for the three dynamic measures are averaged by node, creating a measure called 'DynAvg' analogously to the averaged dynamic impact measure I. This serves as the basis for labelling nodes as having high or low dynamic impact. Nodes are classified as having high impact if their z-score value is above the median of the distribution of 'DynAvg'. This median and the resulting dynamic labels are determined anew for each test set.
To determine reclassification accuracy, normalisation is performed across all nodes without the use of a separate test set.

In the following Figures $S1$ and $S2$, the results of multiple classification algorithms are shown, presenting the best combination of static features for the prediction of this dynamics-based label. Here, every sample corresponds to a single node, yielding a total of 684 samples and 8 static measures. For all algorithms, both a 10x10 as well as a leave-one-subset-out (LOSS, leaving out one network) cross-validation have been performed.
We ran classification using all possible combinations of the graph-based features over all nodes of all networks. For classification we used kNN with $k = \{1, 3, 5\}$, a random forest and SVMs with linear and RBF kernels. For the 10x10-CV, results show a cross-validation accuracy ranging between minima of 0.461 and 0.545 across algorithms for the worst-performing feature combination and maxima between 0.691 and 0.749 for the best performing combination. Likewise for the LOSS-CV, cross-validation accuracy ranged from minima of 0.335 to 0.494 while its maxima ranged from 0.665 to 0.722.
Analogously, the reclassification accuracy ranges between minima of 0.469 and 0.569 to maxima between 0.705 and 1.0.

Given the preferential selection of VB and DP among well-performing feature combinations across algorithms, we further focus our investigation on these two measures. We expect a combination of both static features to perform best. This will be compared to the accuracy of the individual measures.

3 Determination of threshold

Depending on whether a gene is correctly classified by our static ranking as having high/low impact or not, measures of sensitivity and specificity are used to quantify rates of false positives (FP) and false negatives (FN) in relation to true positives or negatives (TP or TN).
These are defined as

$$\text{sensitivity} = \frac{\#\text{TP}}{\#(\text{TP} + \text{FN})}$$

$$= \frac{\text{high static \& dynamic impact genes}}{\text{high dynamic impact genes}}$$

$$\text{specificity} = \frac{\#\text{TN}}{\#(\text{TN} + \text{FP})}$$

$$= \frac{\text{low static \& dynamic impact genes}}{\text{low dynamic impact genes}}$$

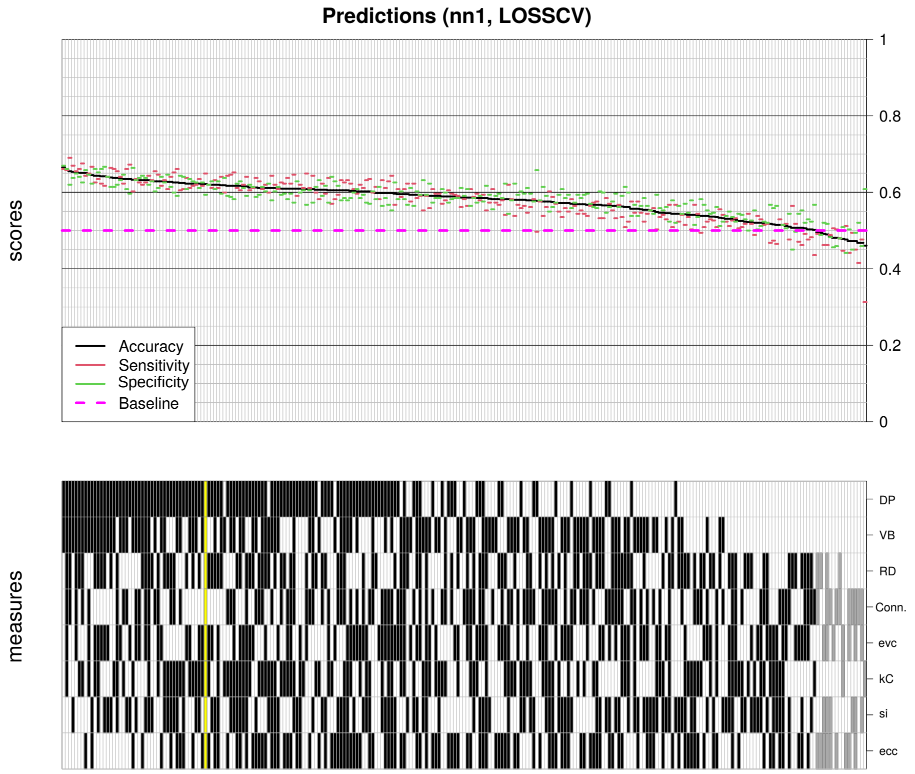
Predictions (nn1, LOSSCV)
scores
1
0.8
0.6
0.4
0.2
0
Accuracy
Sensitivity
Specificity
Baseline
measures
DP
VB
RD
Conn.
evc
kC
si
ecc

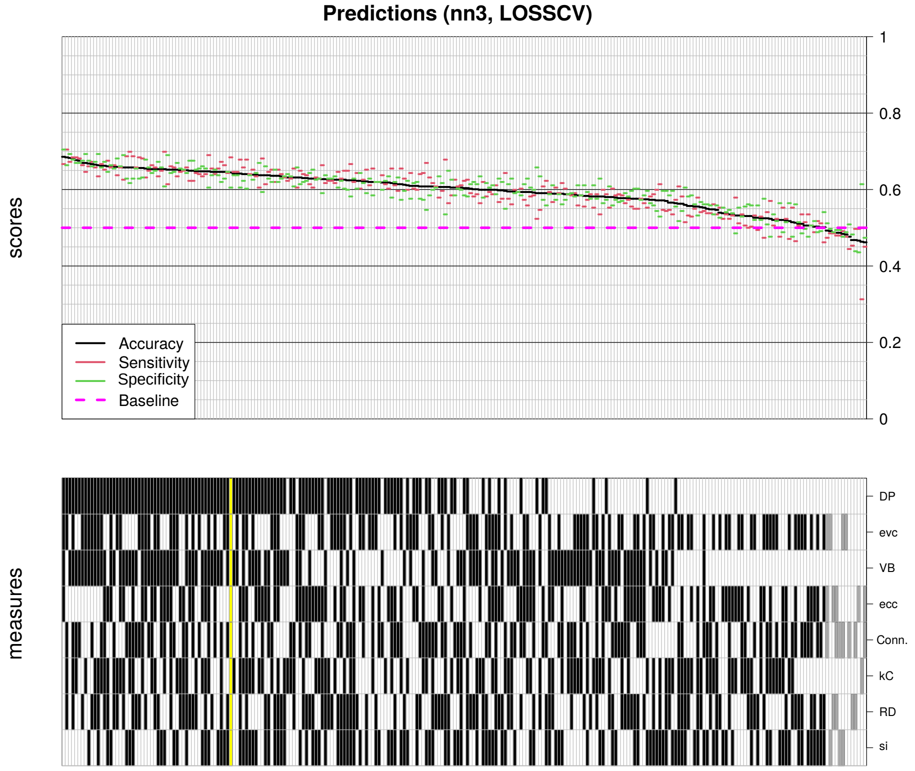
Predictions (nn3, LOSSCV)
scores
1
0.8
0.6
0.4
0.2
0
Accuracy
Sensitivity
Specificity
Baseline
measures
DP
evc
VB
ecc
Conn.
kC
RD
si

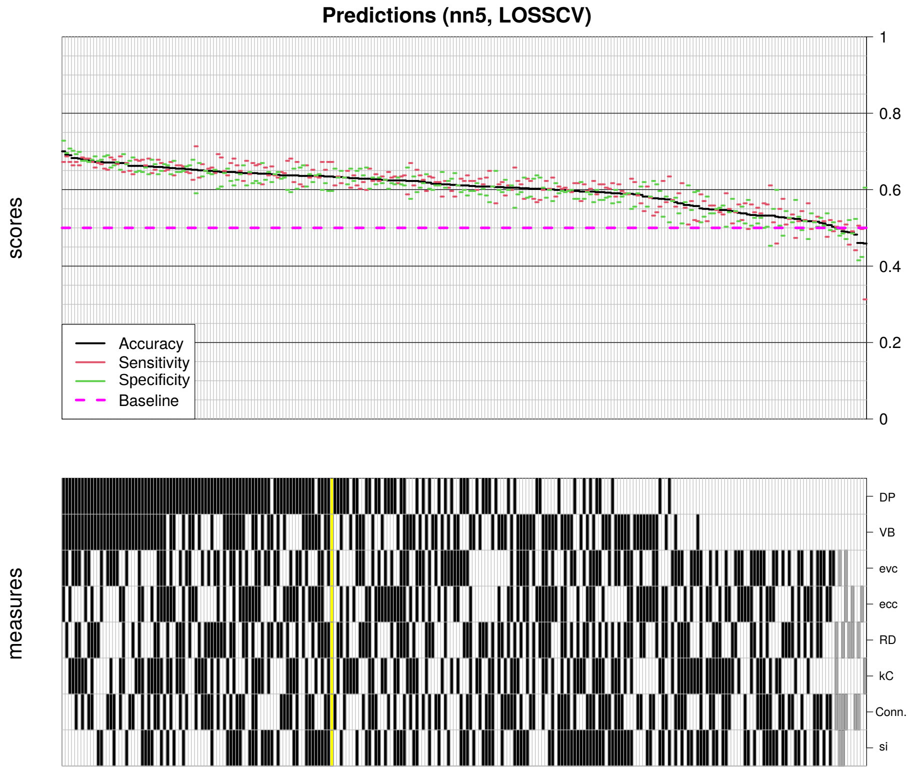
Predictions (nn5, LOSSCV)
scores
Accuracy
Sensitivity
Specificity
Baseline
1
0.8
0.6
0.4
0.2
0
measures
DP
VB
evc
ecc
RD
kC
Conn.
si

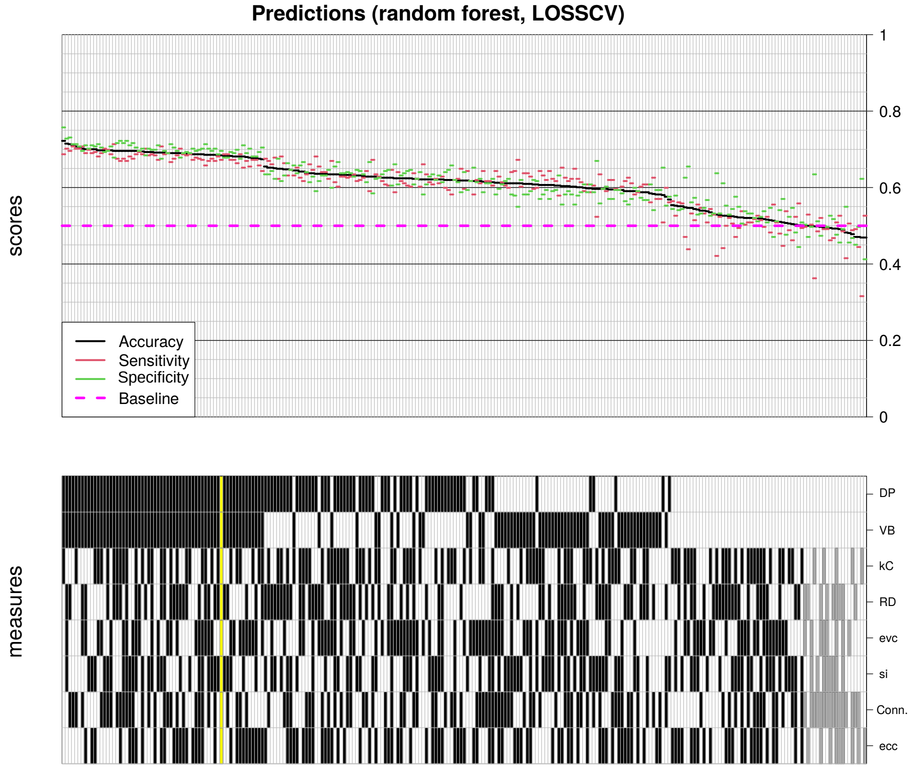
Predictions (random forest, LOSSCV)
scores
Accuracy
Sensitivity
Specificity
Baseline
1
0.8
0.6
0.4
0.2
0
measures
DP
VB
kC
RD
evc
si
Conn.
ecc

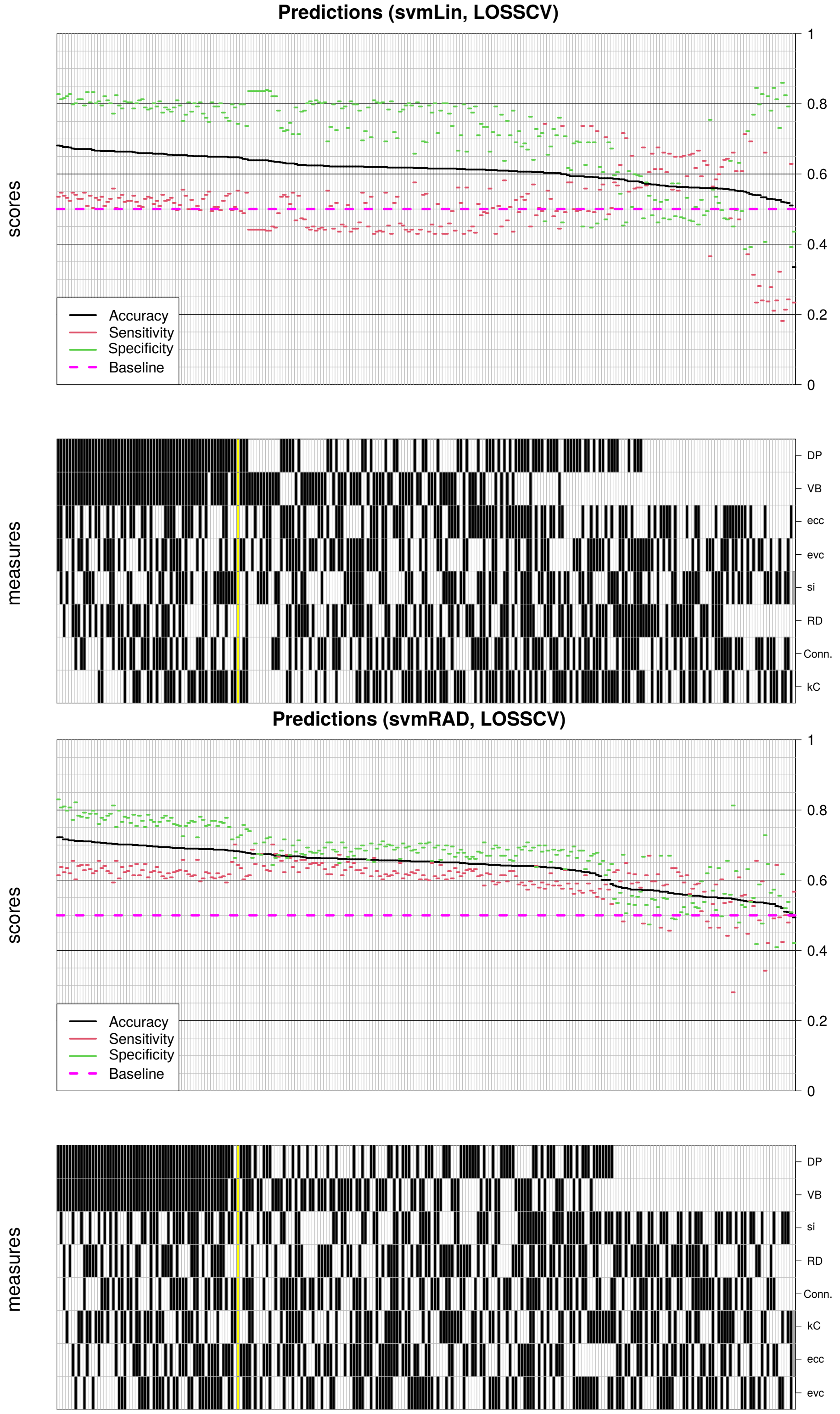

Figure S1: Performance of various algorithms for predicting the dynamics-based binary label from combinations of static features. Every sample corresponds to a single node with a total of 684 samples across networks. The shown algorithms include kNN (with $k = \{1, 3, 5\}$), a random forest (RF) approach as well as an SVM with a linear and RBF-kernel. In all cases, leave-one-subset-out cross-validation (LOSS-CV) has been performed. The static measures are sorted from best to worst-performing (top to bottom).

Predictions (nn1, 10x10CV)

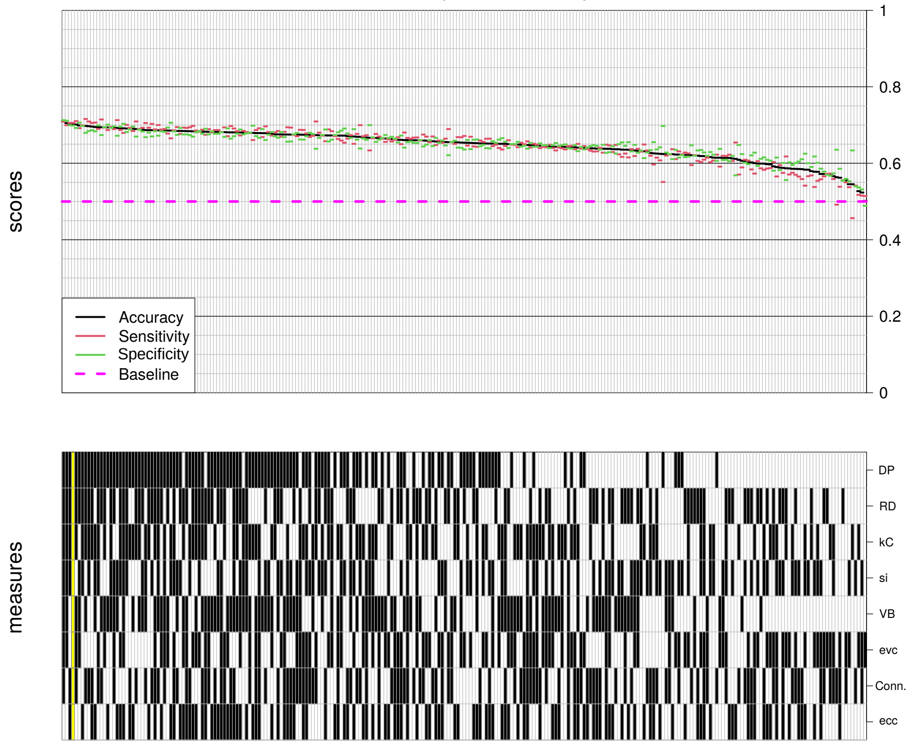

Predictions (nn3, 10x10CV)

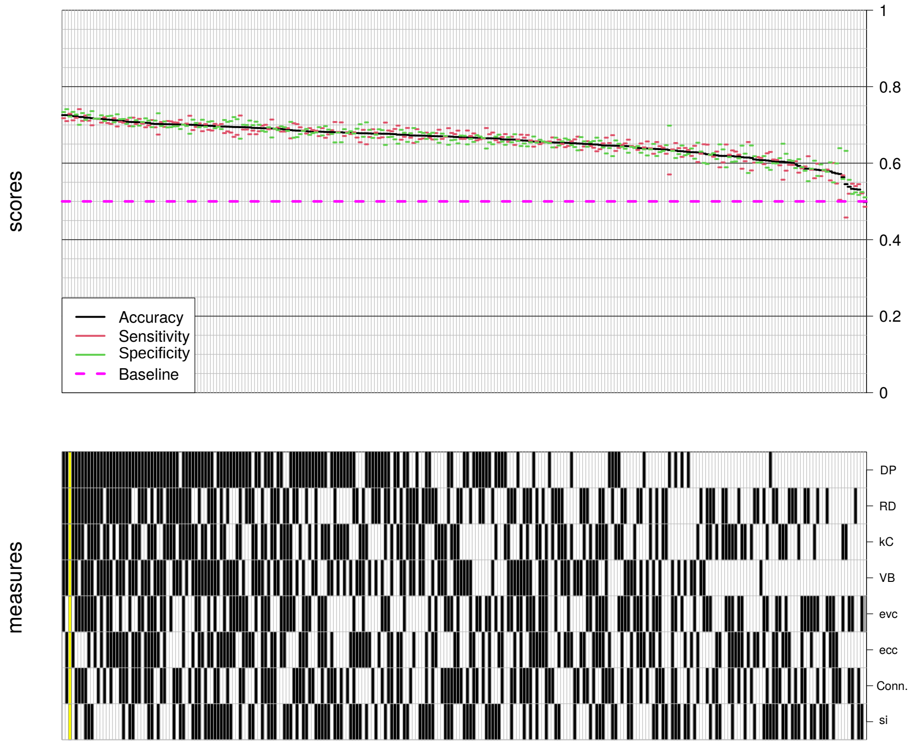

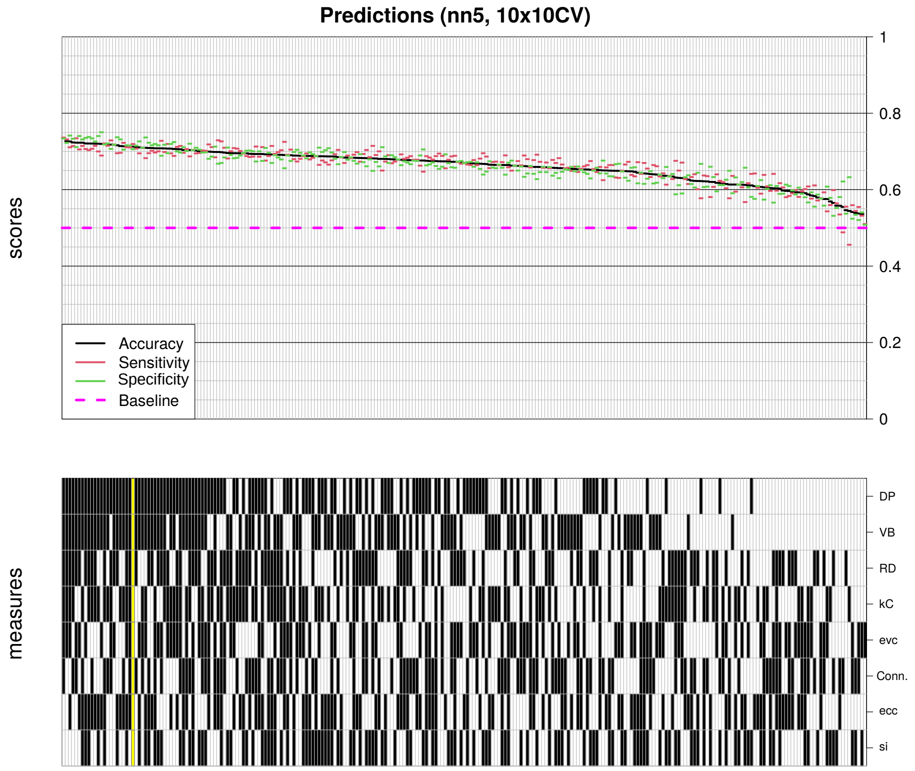
Predictions (nn5, 10x10CV)
scores
1
0.8
0.6
0.4
0.2
0
Accuracy
Sensitivity
Specificity
Baseline
measures
DP
VB
RD
kC
evc
Conn.
ecc
si

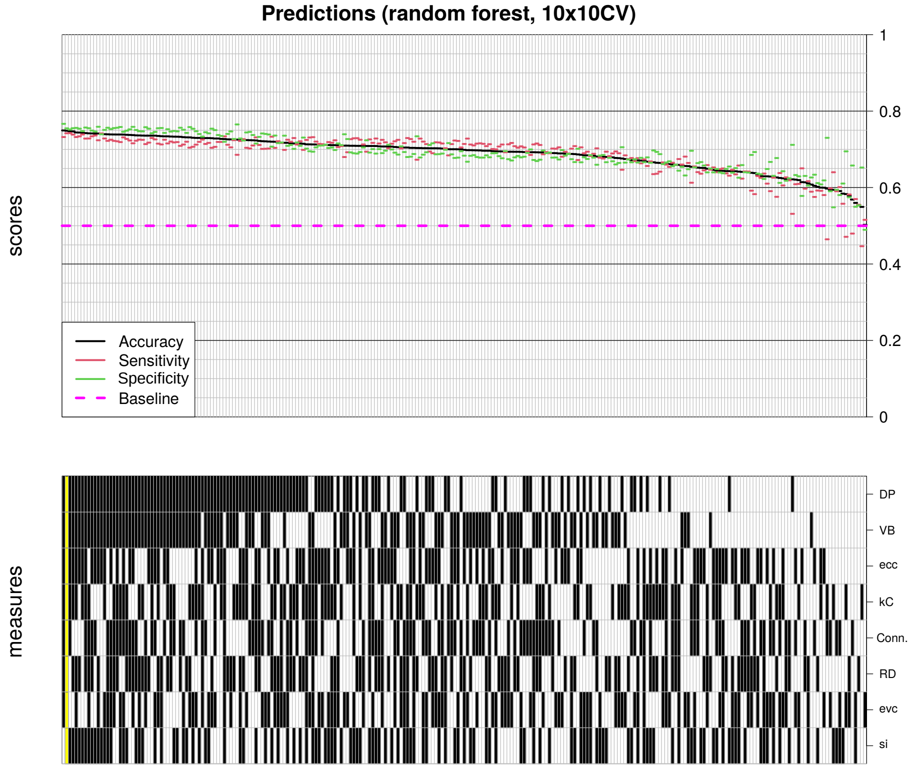
Predictions (random forest, 10x10CV)
scores
1
0.8
0.6
0.4
0.2
0
Accuracy
Sensitivity
Specificity
Baseline
measures
DP
VB
ecc
kC
Conn.
RD
evc
si

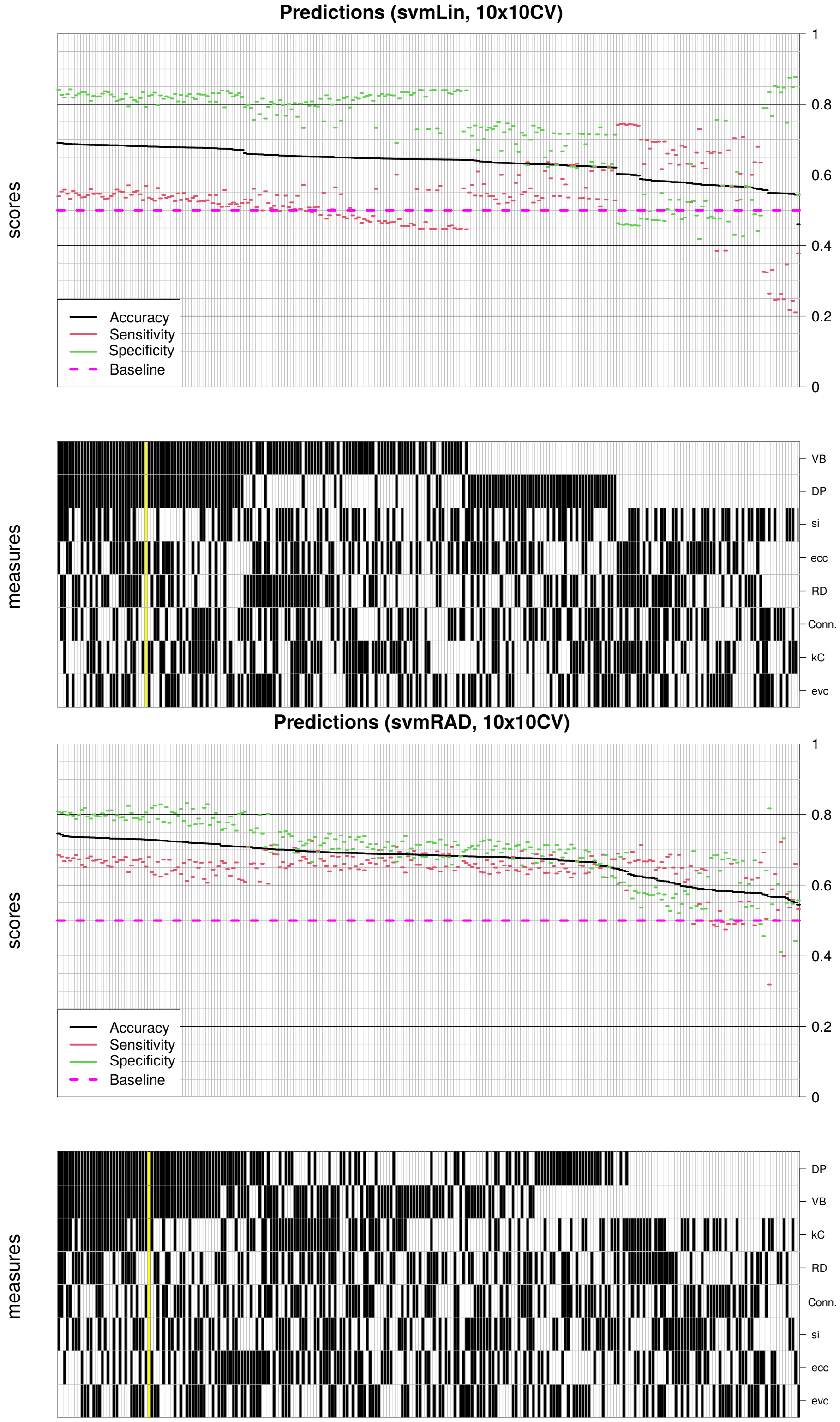

Figure S2: Performance of various algorithms for predicting the dynamics-based binary label from combinations of static features. Every sample corresponds to a single node with a total of 684 samples across networks. The shown algorithms include kNN (with $k = \{1, 3, 5\}$), a random forest (RF) approach as well as an SVM with a linear and RBF-kernel. In all cases, 10x10 cross-validation has been performed. The static measures are sorted from best to worst-performing (top to bottom).

4 Sensitivity-specificity curves of averaged dynamic impact for all static measures

Figure S3 shows the mean and standard deviations for sensitivity and specificity across networks for all four static measures (VB, DP, VB∪DP and VB∩DP).

For every T, the nodes are ranked according to their scores on VB and DP. The top $T' = \left\lceil \frac{T}{100} n \right\rceil$ nodes are then selected and compared to the corresponding selection based on a dynamic measure. For VB∪DP, the top T' nodes are chosen for both VB and DP and the resulting sets are unified. If a node is chosen by both measures, their rankings are averaged. If it is only selected by one of the two, its ranking according to this measure is used. In case of VB∩DP, the intersection of the top T' nodes in VB and DP is formed, averaging their rankings.

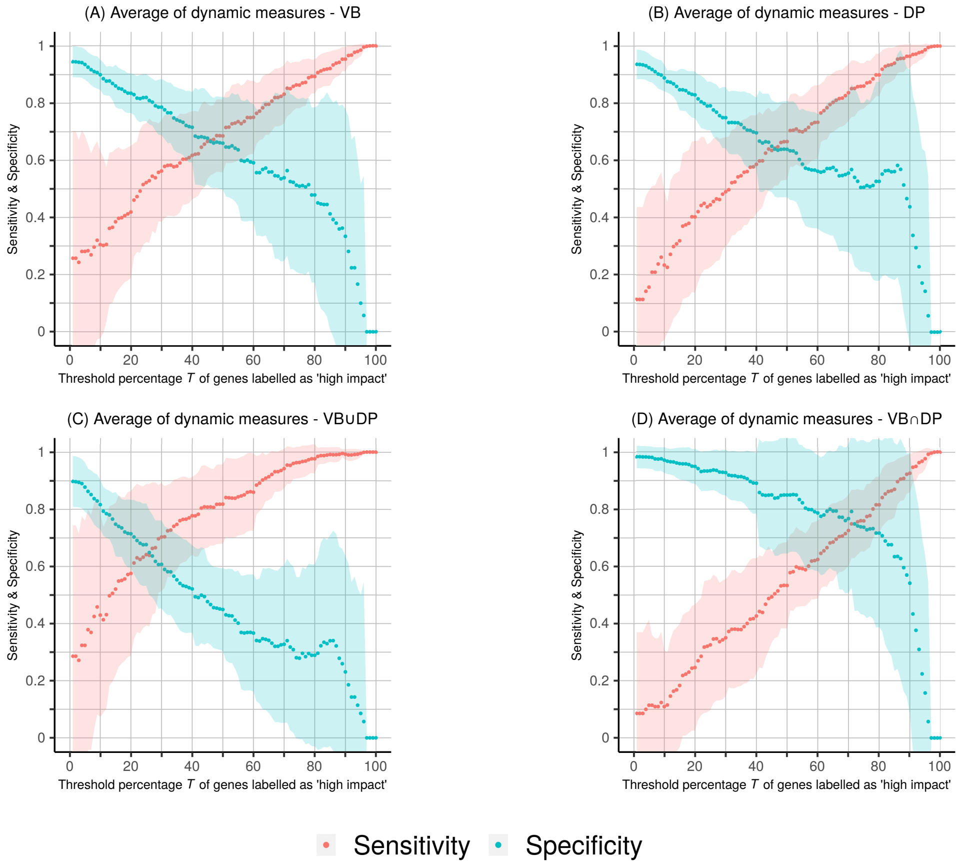

Figure S3: The means of the sensitivity and specificity across 35 biological networks using different thresholds for labelling genes as having high impact. The static measures VB, DP and their combinations are compared to the the average of the three dynamic measures of attractor gain, attractor loss and minimal Hamming distance. The measures VB and DP show sensitivity values of 0.669 and 0.643 at $T = 46\%$. The union VB∪DP has a value of 0.663 at $T = 27\%$ while the intersection VB∩DP has a sensitivity of 0.756 at $T = 73\%$. At thresholds of $T = \{46\%, 46\%, 27\%, 73\%\}$, rounding up to select $T' = \left\lceil \frac{T}{100} n \right\rceil$ nodes and given the operation of taking a union or intersection of selected nodes in VB and DP, these measures yield a total of 333 (48.7%), 333 (48.7%), 305 (44.6%), and 424 (62.0%) nodes respectively across networks. Given the superior performance of VB∩DP, we further focus on this measure, classifying selected nodes as PM (gatekeepers) or NM.

5 Sensitivity-specificity curves for individual dynamic measures

Figure S4 shows sensitivity-specificity values for any given threshold for the three individual dynamic measures of attractor loss, attractor gain and Hamming distance. The obtained thresholds are demonstrated to vary by 1-3% for the value of T at which sensitivity and specificity are equal, indicating that the results are independent of the chosen dynamic measure.

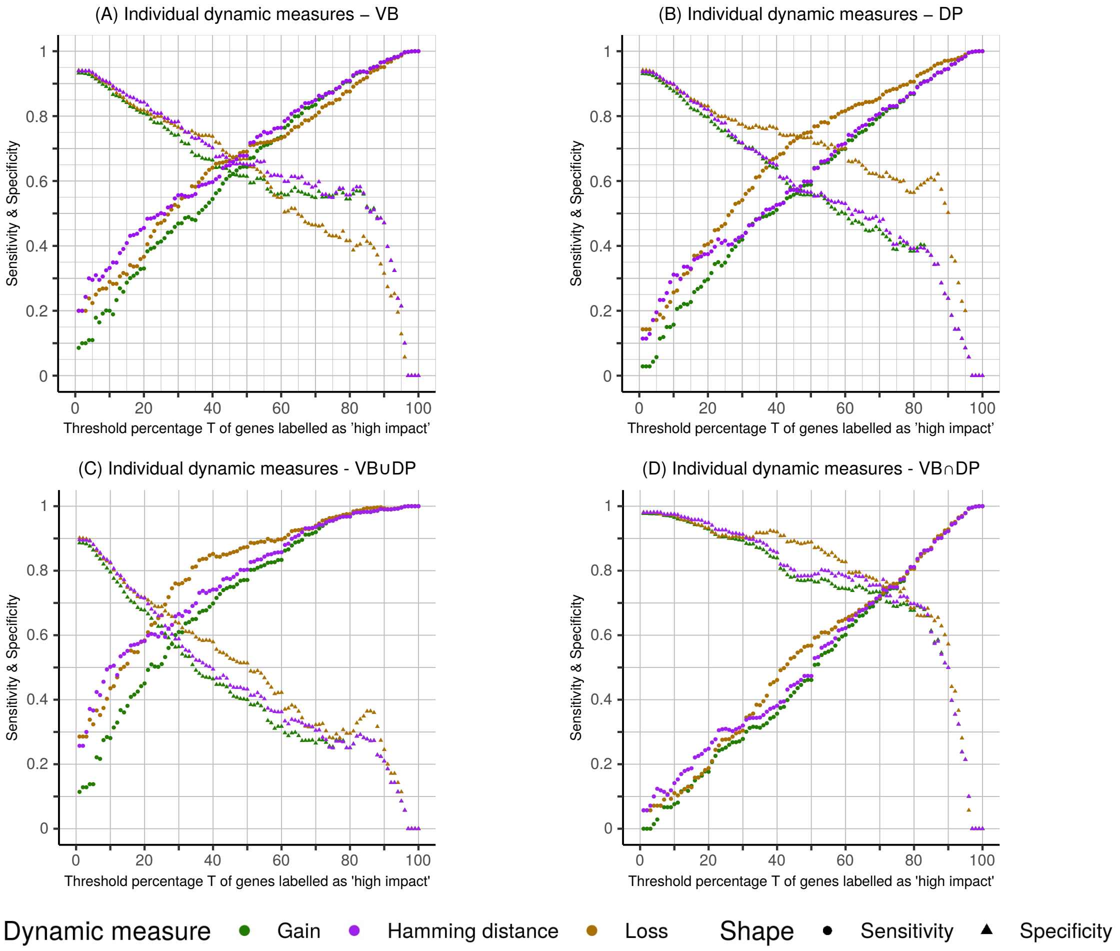

Figure S4: The means of the sensitivity and specificity across 35 biological networks using different thresholds for labelling genes as having high impact. The static measures VB, DP and their combinations are compared to the dynamic measures of attractor gain, attractor loss as well as to minimal Hamming distances. For the measure of attractor gain, the values for sensitivity and specificity are balanced for thresholds of $T = \{46, 46, 29, 72\}$ for (A) VB alone, (B) DP alone, (C) the union of VB and DP and (D) the intersection of VB and DP respectively. For the measures of attractor loss and minimal Hamming distance, the corresponding thresholds are $T = \{47\%, 47\%, 26\%, 74\%\}$ and $T = \{46\%, 46\%, 27\%, 72\%\}$ respectively.

6 Bootstrap test for selection threshold

In order to determine the stability of a chosen threshold for the selected static measure, a bootstrap analysis is performed. That is, out of the $N = 35$ networks, a random sample of $N' = 35$ networks is chosen (with replacement) and the selection threshold of the static measure is recalculated for the three individual dynamic measures as well as their average. This process is repeated 10^4 times.

The distributions of obtained thresholds are shown in Figure S5. For the average of the three dynamic measures, a median threshold of 73.0% was obtained with an interquartile range for 4.0%. This indicates that the chosen threshold value is not dependent on the data set used for the calculation of sensitivity and specificity.

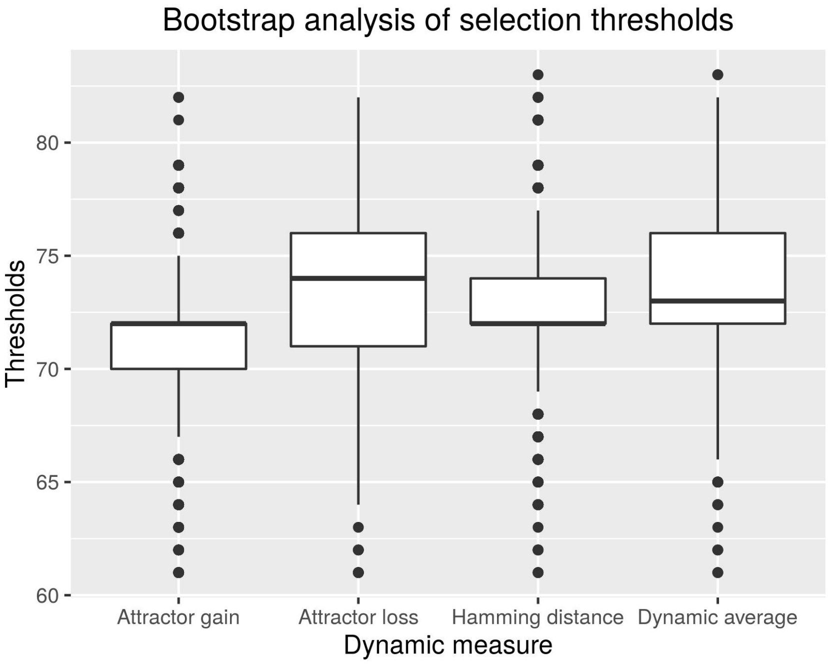

Figure S5: The distribution of selection thresholds where sensitivity and specificity are equal for every dynamic measure as well as their average, obtained using a bootstrap analysis with 10^4 repetitions. For the average of dynamic measures, the median of the distribution is 73.0% with an interquartile range of 4.0%.

7 Accuracy of selection by network size

Figure $S6$ shows the overlap of the sets of nodes ranking highest on static measures and the average of dynamic measures at the determined threshold of $T = 73\%$, normalised by the number of dynamically relevant nodes.

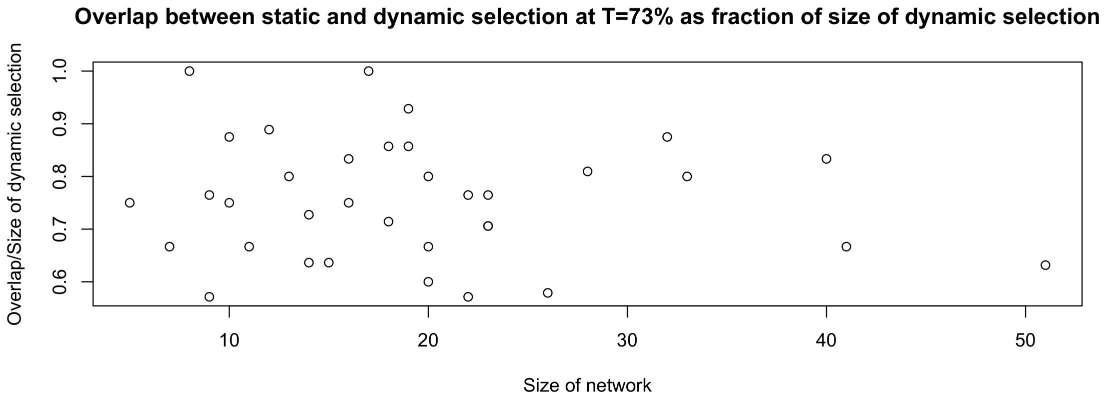

Figure S6: Overlap of sets determined by selecting the highest ranking nodes according to VB and DP and dynamic measures at the previously determined selection threshold of $T = 73\%$ (y-axis) versus network size (x-axis).

8 Case study: Classification of nodes

8.1 Network by Cohen et al.

Among the 32 nodes in the network presented by (Cohen *et al.*, 2015), the following classifications are obtained using the measure of VB∩DP at $T = 73\%$:

PM(Gatekeepers) (11 nodes, 34.4%): TWIST1, Migration, CDH1, ERK, TGFbeta, CDH2, CTNNB1, miR203, ZEB2, miR200, AKT1
NM (10 nodes, 31.3%): p53, p63, p73, SNAI2, miR34, NICD, AKT2, SNAI1, ZEB1, SMAD
NS (11 nodes, 34.4%): DNAdamage, ECM, p21, Apoptosis, EMT, GF, Invasion, CellCycleArrest, DKK1, Metastasis, VIM
PM and NM nodes are sorted in order of highest to lowest positive/negative mismatch.

8.2 Network by Méndez-López et al.

Likewise, the 9 nodes in the network of (Méndez-López *et al.*, 2017) are classified as:

PM(Gatekeepers) (2 nodes, 22.2%): ESE2, p16
NM (3 nodes, 33.3%): p53, Snai2, NFkB
NS (4 nodes, 44.4%): Cyclin, TELase, Rb, E2F

8.3 Network by Krumsiek et al.

For the network presented by (Krumsiek *et al.*, 2011), the 11 nodes are classified as:

PM(Gatekeepers) (5 nodes, 45.5%): CEBPa, FLI1, PU1, GATA2, Gfi1
NM (2 nodes, 18.2%): GATA1, EgrNab
NS (4 nodes, 36.4%): FOG1, EKLF, SCL, cJun

9 Run time differences

An advantage of the use of static methods for intervention target screening is that their run times are orders of magnitude smaller than for the corresponding attractor measures.
Across networks, it was found that the suggested static measure of vertex betweenness could be calculated on a time scale of milliseconds while the average time to calculate the determinative power of all nodes in a network was 1.3 seconds using a single core of a desktop computer with a 3.2 GHz Intel Core i5-6500 processor and 16 GB of memory. For comparison, the presented dynamic measures required on average 13.3 seconds to calculate.
For a larger network consisting of 131 nodes and 313 edges (Madrahimov *et al.*, 2013), determining the system's set of attractors using BoolNet's `getAttractors(SBML, method="sat.exhaustive")` function required 48.5 hours of run time. Given this size, the state transition graph has a size of $2^{131} \approx 10^{39}$ nodes. This prohibits the use of the proposed dynamic measures which require a repeated execution of this function. If the expression of a single gene is fixed, the size of the state transition graph will be cut in half. However, two modifications (OE and KO) are performed for every gene. Thus the run time of the dynamic measures is estimated at $131 \cdot 48.5 = 6353.5$ hours ≈ 265 days for this network.
In contrast, the measure DP could be determined in 84.8 seconds for this system while VB was still computable in milliseconds.

10 Analysis of selected nodes

10.1 Classification for different z-score definitions

We quantified the connectivity C of a gene g using a z-score for their total in- and output degree δ given as

$$C_g = \frac{\delta_g - \bar{\delta}}{\sigma} \tag{5}$$

Alternative, a robust z-score C'_g based on the median can be used, which is defined as

$$\text{MAD} = \text{median}(|\delta_g - \text{median}(\delta)|)$$
$$C'_g = \frac{\delta_g - \text{median}(\delta)}{\text{MAD}}$$

The following Table S2 shows the classification of nodes for all 35 networks for both of these definitions. We found that the choice of z-score does not affect which nodes are classified as PM (gatekeeper) or NM. However, for the networks (Méndez and Mendoza, 2016) and (Mendoza and Xenarios, 2006), the MAD became zero, which is why the robust z-score could not be calculated in these cases.

Authors and Year	z-score			Robust z-score		
	PM	NM	NS	PM	NM	NS
(Azpeitia *et al.*, 2013)	5	4	5	5	4	5
(Brandon *et al.*, 2015)	11	4	7	11	4	7
(Calzone *et al.*, 2010)	8	10	10	8	10	10
(Cohen *et al.*, 2015)	11	10	11	11	10	11
(Dahlhaus *et al.*, 2016)	8	5	10	8	5	10
(Davila-Velderrain *et al.*, 2015)	4	4	5	4	4	5
(Enciso *et al.*, 2016)	7	7	12	7	7	12
(Fauré *et al.*, 2006)	2	4	4	2	4	4
(García-Gómez *et al.*, 2017)	5	6	5	5	6	5
(Giacomantonio and Goodhill, 2010)	2	2	1	2	2	1
(Gupta *et al.*, 2007)	3	6	7	3	6	7
(Herrmann *et al.*, 2012)	5	3	7	5	3	7
(Irons, 2009)	6	5	7	6	5	7
(Klamt *et al.*, 2006)	17	8	15	17	8	15
(Krumsiek *et al.*, 2011)	5	2	4	5	2	4
(MacLean and Studholme, 2010)	0	5	2	0	5	2
(Mai and Liu, 2009)	13	8	20	13	8	20
(Marques-Pita and Rocha, 2013)	7	6	4	7	6	4
(Martinez-Sanchez *et al.*, 2015)	3	5	4	3	5	4
(Méndez and Mendoza, 2016)	10	5	7	-	-	7
(Méndez-López *et al.*, 2017)	2	3	4	2	3	4
(Mendoza and Xenarios, 2006)	8	5	10	-	-	10
(Meyer *et al.*, 2017)	20	11	20	20	11	20
(Orlando *et al.*, 2008)	2	4	3	2	4	3
(Ortiz-Gutiérrez *et al.*, 2015)	4	5	5	4	5	5
(Ríos *et al.*, 2015)	5	7	7	5	7	7
(Saadatpour *et al.*, 2011)	8	5	5	8	5	5
(Sahin *et al.*, 2009)	4	7	9	4	7	9
(Sankar *et al.*, 2011)	8	5	7	8	5	37
(Siegle *et al.*, 2018)	7	6	10	7	6	10
(Sridharan *et al.*, 2012)	7	6	6	7	6	6
(Sun *et al.*, 2014)	3	3	2	3	3	2
(Thakar *et al.*, 2012)	12	9	12	12	9	12
(Todd and Helikar, 2012)	3	7	10	3	7	10
(Yousefi and Dougherty, 2013)	2	5	3	2	5	3

Table S2: Authors of the 35 selected networks, and numbers of nodes assigned to a given class used both an average and standard deviation based z-score as well as a robust median and MAD based z-score.

10.2 Comparison of selection based on static measures to other methods of capturing dynamic relevance

We compared our method to other common methods to detect nodes with high dynamic impact. These other methods include which are canalysing variables for some Boolean functions in their network (Murrugarra and Dimitrova, 2015), nodes making up the feedback vertex set (FVS) of a network (Zañudo *et al.*, 2017), as well as nodes which appear in common network motifs such as feedforward-loops or bifans (Milo *et al.*, 2002; Albergante *et al.*, 2014).
The following Table S3 shows the number of nodes across 684 nodes in all networks which function as canalysing nodes for at least F Boolean functions in their respective network. These results are compared to the nodes selected by our method (PM and NM) and the set of nodes which was not selected by out method.
A distribution of nodes in the FVS across the classes PM, NM and NS across networks is shown in Figure S8.
Given their relevance and stability as mentioned by (Albergante *et al.*, 2014), we investigated feedforward loops (both the coherent C1FFL and the incoherent I1FFL) as well as the bifan. We counted the number of motifs of a given type a node participates in and plotted the distribution of these occurrences depending on their classification as shown in Figure S7. Across networks, there were a total of 1086 C1FFLs, 112 I1FFLs and 1197 bifans.

F	Total nodes	PM(Gatekeepers) selected	NM	NS non-selected
1	543	222	179	142
2	283	119	108	56
3	137	55	62	20
4	71	26	38	7
5	34	9	22	3
6	17	7	10	0
7	9	3	6	0
8	6	1	5	0
9	6	1	5	0
10	4	1	3	0
11	1	0	1	0

Table S3: Occurrence of nodes which canalyse at least F Boolean functions in their respective network across all 684 nodes, as well as their distribution across the classes PM (positive mismatch, gatekeeper), NM (negative mismatch), NS (non-selected) and hubs. As F increases, the remaining canalysing nodes tend to belong to the more highly connected NM class.

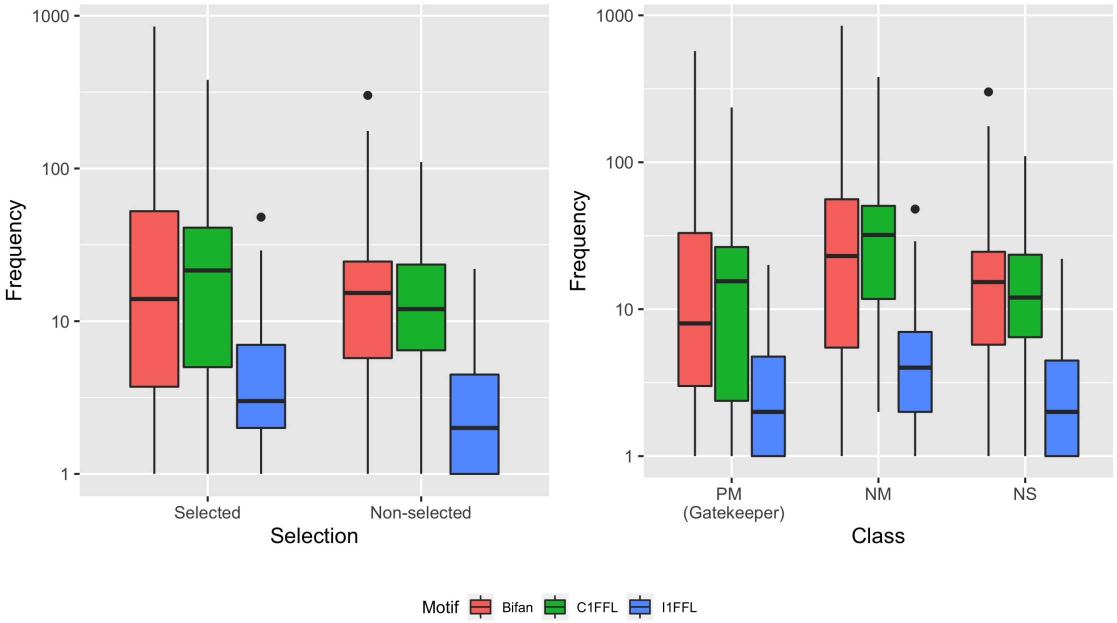

Figure S7: Number of motifs by network which selected (PM or NM) or non-selected (NS) nodes participate in, across all 35 networks (top). The number of motifs is further separated by class (bottom). Frequency is plotted on a logarithmic scale.

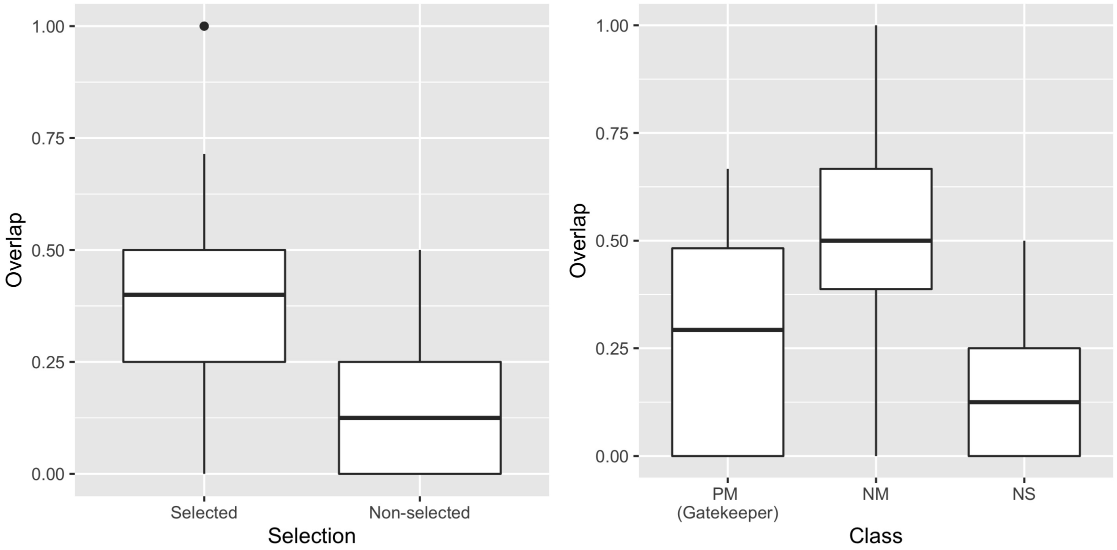

Figure S8: Overlap of FVS nodes with selected nodes (either PM(Gatekeepers) or NM) versus non-selected nodes (NS) by network over all 35 networks, normalised by the size of the FVS (left). Distribution across all classes PM/Gatekeepers, NM and NS (right). FVS is calculated using the feedback_vertex_set function in SageMath9.2 (Stein and Joyner, 2005).

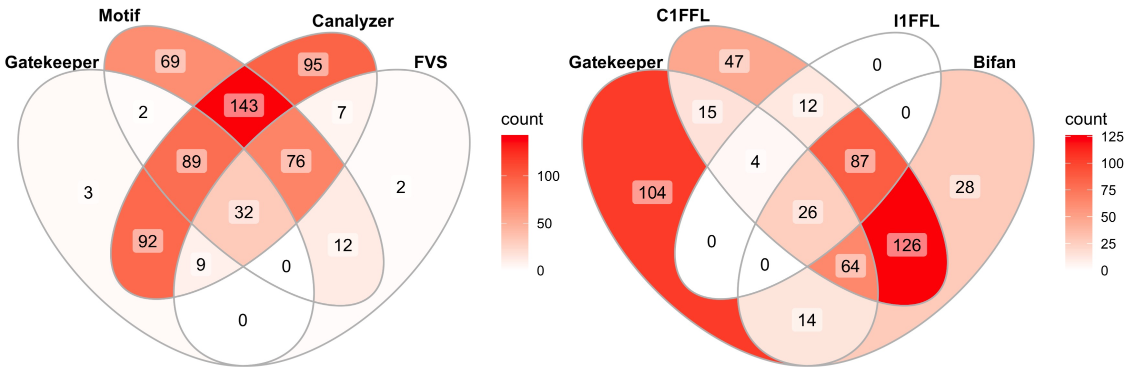

Figure S9: Venn diagrams showing the overlap between gatekeepers (227 in total), FVS nodes (138 in total), nodes which canalyse at least one Boolean function in their network (543 in total) and nodes which participate in at least one motif (423 in total) (left). The participation in motifs is further visualised as follows: 381 nodes participate in at least one C1FFL motif, 129 in at least one I1FFL and 345 in at least one Bifan (right).

10.3 Biological interpretation of selected nodes

The STRING (Szklarczyk *et al.*, 2016) analysis was performed by parsing our networks into the STRING database and checking for co-expressed genes, with the high confidence given by the tool (confidence level of high, 0.7, see Table S4)

class	co-occurence	co-expression
PM (Gatekeepers)	1	1
NM	0	3
NS	0	1

Table S4: Number of co-occuring or co-expressed compounds in each class of the predicted nodes across all 35 measured networks according.

Next, we used cBioportal to screen for mutually exclusive pairings compounds across all 22 human networks among the set of analysed networks.

Network reference	Mutually excl. pair	Type	p-value
(Klamt *et al.*, 2006)	BRAS - KRAS	PM-PM	< 0.001
	KRAS - NRAS	PM-PM	< 0.001
(Sridharan *et al.*, 2012)	KRAS-NRAS	PM-PM	< 0.001
	NRAS-PI3KCA	PM-PM	0.017

Table S5: List of mutually exclusive pairs individuated by cBioportal (Gao *et al.*, 2013) analysis on human networks.

References

Albergante, L. *et al.* (2014). Buffered qualitative stability explains the robustness and evolvability of transcriptional networks. *Elife*, **3**, e02863.

Azpeitia, E. *et al.* (2013). Finding missing interactions of the arabidopsis thaliana root stem cell niche gene regulatory network. *Frontiers in Plant Science*, **4**, 110.

Brandon, M. *et al.* (2015). Iron acquisition and oxidative stress response in aspergillus fumigatus. *BMC Systems Biology*, **9**(1), 19.

Calzone, L. *et al.* (2010). Mathematical modelling of cell-fate decision in response to death receptor engagement. *PLoS Computational Biology*, **6**(3).

Clauset, A. *et al.* (2009). Power-law distributions in empirical data. *SIAM Review*, **51**(4), 661–703.

Cohen, D. P. *et al.* (2015). Mathematical Modelling of Molecular Pathways Enabling Tumour Cell Invasion and Migration. *PLoS Computational Biology*, **11**(11), e1004571.

Dahlhaus, M. *et al.* (2016). Boolean modeling identifies Greatwall/MASTL as an important regulator in the AURKA network of neuroblastoma. *Cancer Letters*, **371**(1), 79–89.

Davila-Velderrain, J. *et al.* (2015). Reshaping the epigenetic landscape during early flower development: induction of attractor transitions by relative differences in gene decay rates. *BMC Systems Biology*, **9**(1), 20.

Enciso, J. *et al.* (2016). Modeling the pro-inflammatory tumor microenvironment in acute lymphoblastic leukemia predicts a breakdown of hematopoietic-mesenchymal communication networks. *Frontiers in Physiology*, **7**, 349.

Fauré, A. *et al.* (2006). Dynamical analysis of a generic boolean model for the control of the mammalian cell cycle. *Bioinformatics*, **22**(14), e124–e131.

Freeman, L. C. (1977). A Set of Measures of Centrality based on Betweenness. *Sociometry*, pages 35–41.

Gao, J. *et al.* (2013). Integrative analysis of complex cancer genomics and clinical profiles using the cbioportal. *Science signaling*, **6**(269), pl1–pl1.

García-Gómez, M. L. *et al.* (2017). A dynamic genetic-hormonal regulatory network model explains multiple cellular behaviors of the root apical meristem of arabidopsis thaliana. *PLoS Computational Biology*, **13**(4), e1005488.

Giacomantonio, C. E. and Goodhill, G. J. (2010). A boolean model of the gene regulatory network underlying mammalian cortical area development. *PLoS Computational Biology*, **6**(9).

Giatsidis, C. *et al.* (2013). D-cores: measuring collaboration of directed graphs based on degeneracy. *Knowledge and Information Systems*, **35**(2), 311–343.

Gillespie, C. (2015). PoweRlaw: analysis of heavy tailed distributions. *R package version 0.30. 0, URL http://CRAN. R-project. org/package= poweRlaw*.

Guimera, R. and Amaral, L. A. N. (2005). Functional cartography of complex metabolic networks. *Nature*, **433**(7028), 895–900.

Gupta, S. *et al.* (2007). Boolean network analysis of a neurotransmitter signaling pathway. *Journal of Theoretical Biology*, **244**(3), 463–469.

Hage, P. and Harary, F. (1995). Eccentricity and centrality in networks. *Social Networks*, **17**(1), 57–63.

Heckel, R. *et al.* (2013). Harmonic analysis of Boolean networks: Determinative power and perturbations. *EURASIP Journal on Bioinformatics and Systems Biology*, **2013**(1), 6.

Helikar, T. *et al.* (2012). The Cell Collective: Toward an open and collaborative approach to systems biology. *BMC Systems Biology*, **6**(1), 96.

Herrmann, F. *et al.* (2012). A Boolean Model of the Cardiac Gene Regulatory Network Determining First and Second Heart Field Identity. *PLoS One*, **7**(10), e46798.

Irons, D. (2009). Logical analysis of the budding yeast cell cycle. *Journal of Theoretical Biology*, **257**(4), 543–559.

Klamt, S. *et al.* (2006). A methodology for the structural and functional analysis of signaling and regulatory networks. *BMC Bioinformatics*, **7**(1), 56.

Klein, D. J. and Randić, M. (1993). Resistance distance. *Journal of Mathematical Chemistry*, **12**(1), 81–95.

Krumsiek, J. *et al.* (2011). Hierarchical differentiation of myeloid progenitors is encoded in the transcription factor network. *PloS One*, **6**(8).

MacLean, D. and Studholme, D. J. (2010). A boolean model of the pseudomonas syringae hrp regulon predicts a tightly regulated system. *PloS One*, **5**(2).

Madrahimov, A. *et al.* (2013). Dynamics of Influenza Virus and Human Host Interactions During Infection and Replication Cycle. *Bulletin of Mathematical Biology*, **75**(6), 988–1011.

Mai, Z. and Liu, H. (2009). Boolean network-based analysis of the apoptosis network: Irreversible apoptosis and stable surviving. *Journal of Theoretical Biology*, **259**(4), 760–769.

Marques-Pita, M. and Rocha, L. M. (2013). Canalization and control in automata networks: Body segmentation in drosophila melanogaster. *PloS One*, **8**(3).

Martinez-Sanchez, M. E. *et al.* (2015). A minimal regulatory network of extrinsic and intrinsic factors recovers observed patterns of cd4+ t cell differentiation and plasticity. *PLoS Computational Biology*, **11**(6).

Matache, M. T. and Matache, V. (2016). Logical Reduction of Biological Networks to Their Most Determinative Components. *Bulletin of Mathematical Biology*, **78**(7), 1520–1545.

Méndez, A. and Mendoza, L. (2016). A network model to describe the terminal differentiation of b cells. *PLoS Computational Biology*, **12**(1).

Méndez-López, L. F. *et al.* (2017). Gene regulatory network underlying the immortalization of epithelial cells. *BMC Systems Biology*, **11**(1), 24.

Mendoza, L. and Xenarios, I. (2006). A method for the generation of standardized qualitative dynamical systems of regulatory networks. *Theoretical Biology and Medical Modelling*, **3**(1), 13.

Meyer, P. *et al.* (2017). A model of the onset of the senescence associated secretory phenotype after DNA damage induced senescence. *PLoS Computational Biology*, **13**(12), e1005741.

Milo, R. *et al.* (2002). Network motifs: Simple building blocks of complex networks. *Science*, **298**(5594), 824–827.

Murrugarra, D. and Dimitrova, E. S. (2015). Molecular network control through boolean canalization. *EURASIP Journal on Bioinformatics and Systems Biology*, **2015**(1), 9.

Newman, M. E. (2008). The mathematics of networks. *The New Palgrave Encyclopedia of Economics*, **2**(2008), 1–12.

Orlando, D. A. *et al.* (2008). Global control of cell-cycle transcription by coupled cdk and network oscillators. *Nature*, **453**(7197), 944–947.

Ortiz-Gutiérrez, E. *et al.* (2015). A dynamic gene regulatory network model that recovers the cyclic behavior of arabidopsis thaliana cell cycle. *PLoS Computational Biology*, **11**(9).

Ríos, O. *et al.* (2015). A boolean network model of human gonadal sex determination. *Theoretical Biology and Medical Modelling*, **12**(1), 26.

Rodrigue, J.-P. (2016). *The Geography of Transport Systems*. Taylor & Francis.

Saadatpour, A. *et al.* (2011). Dynamical and structural analysis of a t cell survival network identifies novel candidate therapeutic targets for large granular lymphocyte leukemia. *PLoS Computational Biology*, **7**(11).

Sahin, Ö. *et al.* (2009). Modeling erbb receptor-regulated g1/s transition to find novel targets for de novo trastuzumab resistance. *BMC Systems Biology*, **3**(1), 1.

Sankar, M. *et al.* (2011). A qualitative continuous model of cellular auxin and brassinosteroid signaling and their crosstalk. *Bioinformatics*, **27**(10), 1404–1412.

Shimbel, A. (1951). Applications of matrix algebra to communication nets. *The Bulletin of Mathematical Biophysics*, **13**(3), 165–178.

Shimbel, A. (1953). Structural parameters of communication networks. *The Bulletin of Mathematical Biophysics*, **15**(4), 501–507.

Siegle, L. *et al.* (2018). A Boolean network of the crosstalk between IGF and Wnt signaling in aging satellite cells. *PloS One*, **13**(3).

Sridharan, S. *et al.* (2012). Boolean modeling and fault diagnosis in oxidative stress response. *BMC Genomics*, **13**(6), S4.

Stein, W. and Joyner, D. (2005). Sage: System for algebra and geometry experimentation. *ACM SIGSAM Bulletin*, **39**(2), 61–64.

Sun, M. *et al.* (2014). Regulatory logic and pattern formation in the early sea urchin embryo. *Journal of Theoretical Biology*, **363**, 80–92.

Szklarczyk, D. *et al.* (2016). The STRING database in 2017: quality-controlled protein–protein association networks, made broadly accessible. *Nucleic Acids Research*, page gkw937.

Thakar, J. *et al.* (2012). Network model of immune responses reveals key effectors to single and co-infection dynamics by a respiratory bacterium and a gastrointestinal helminth. *PLoS Computational Biology*, **8**(1).

Todd, R. G. and Helikar, T. (2012). Ergodic sets as cell phenotype of budding yeast cell cycle. *PloS One*, **7**(10).

Yousefi, M. R. and Dougherty, E. R. (2013). Intervention in gene regulatory networks with maximal phenotype alteration. *Bioinformatics*, **29**(14), 1758–1767.

Zañudo, J. G. T. *et al.* (2017). Structure-based control of complex networks with nonlinear dynamics. *Proceedings of the National Academy of Sciences*, **114**(28), 7234–7239.
